# Structure–Activity Relationship Study of Protoberberine Alkaloids Reveals Molecular Planarity as a Key Determinant of Membrane Interaction and Protection from Misfolded Protein Oligomers

**DOI:** 10.1021/acs.jmedchem.6c01682

**Published:** 2026-07-27

**Authors:** Silvia Errico, Alessandra Bigi, Giulia Fani, Monica Ambrosino, Erwan Galmiche, Francesco Bemporad, Michele Vendruscolo, Benedetta Mannini, Fabrizio Chiti

**Affiliations:** † Department of Experimental and Clinical Biomedical Sciences, Section of Biochemistry, 9300 University of Florence, Florence 50134, Italy; ‡ Chimie ParisTech - PSL University, Paris 75005, France; § Centre for Misfolding Diseases, 2152 Yusuf Hamied Department of Chemistry, University of Cambridge, Cambridge CB2 1EW, U.K.

## Abstract

Interaction of berberine (Brb) with cell membranes is crucial for its bioavailability, blood–brain barrier permeation, and neuroprotection against misfolded protein oligomers in neurodegenerative diseases. Although Brb follows Lipinski’s rule of five, the structural drivers of its membrane permeation remain unclear. We combined biophysical analyses on biomimetic liposomes with cell culture assays to evaluate eight natural protoberberine alkaloids (PBAs), assessing membrane interactions and protective effects. While traditional physicochemical descriptors varied minimally across the series, molecular planarity, quantified by non-hydrogen out-of-plane atoms, emerged as the key structural feature determining membrane binding affinity, kinetics, stiffening, and protein-oligomer displacement. These membrane effects correlated with the ability of the compounds to mitigate oligomer-induced calcium influx in cell cultures, an early cytotoxicity marker. ANS fluorescence and circular dichroism spectroscopy showed no direct oligomer–PBA interaction, ruling out this mechanism. These findings establish a rational framework for optimizing the Brb benzylisoquinoline scaffold, indicating that increasing molecular planarity enhances membrane affinity and neuroprotection.

## Graphical abstract

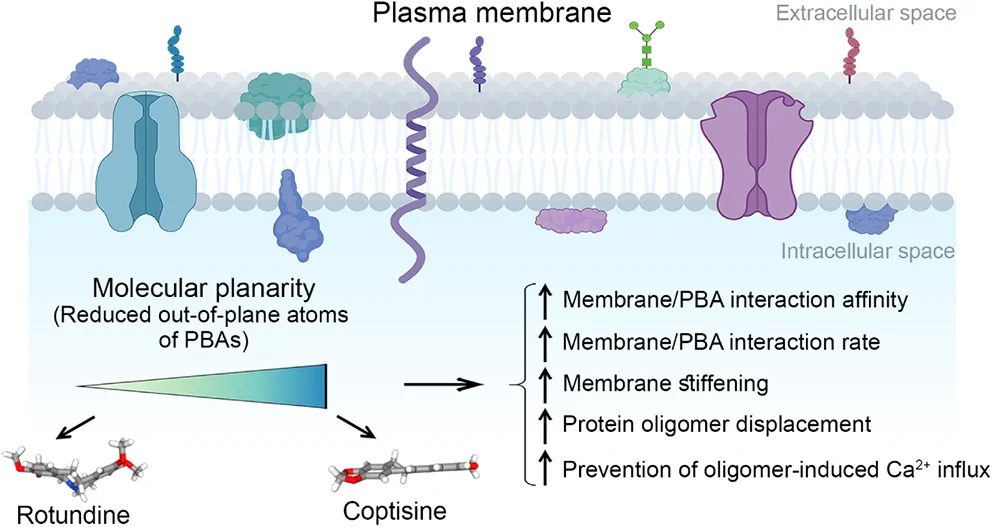

## Introduction

Natural products of plant origin have garnered significant attention for their neuroprotective properties.^1^ Berberine (Brb), a quaternary ammonium salt belonging to the class of benzylisoquinoline alkaloids (Figure 1A), has demonstrated efficacy in various preclinical models of neurodegeneration.^2–7^ These include APP/PS1 transgenic mice as a model of Alzheimer’s disease (AD),^2^ 6-hydroxydopamine-treated SH-SY5Y cells as an in vitro model of Parkinson’s disease (PD),^3^ and SH-SY5Y cells and liposomes as *in vitro* and biomimetic models of AD.^7^ Berberine has well-documented, multifaceted protective effects against neurodegeneration, including antioxidant, anti-inflammatory, and antiapoptotic properties, acetylcholinesterase inhibitory activities, protective effects against amyloid β (Aβ), tau, and α-synuclein pathology, and protection against dysfunction of mitochondria, neurotransmission, and autophagy.^8–10^

**Figure 1:**
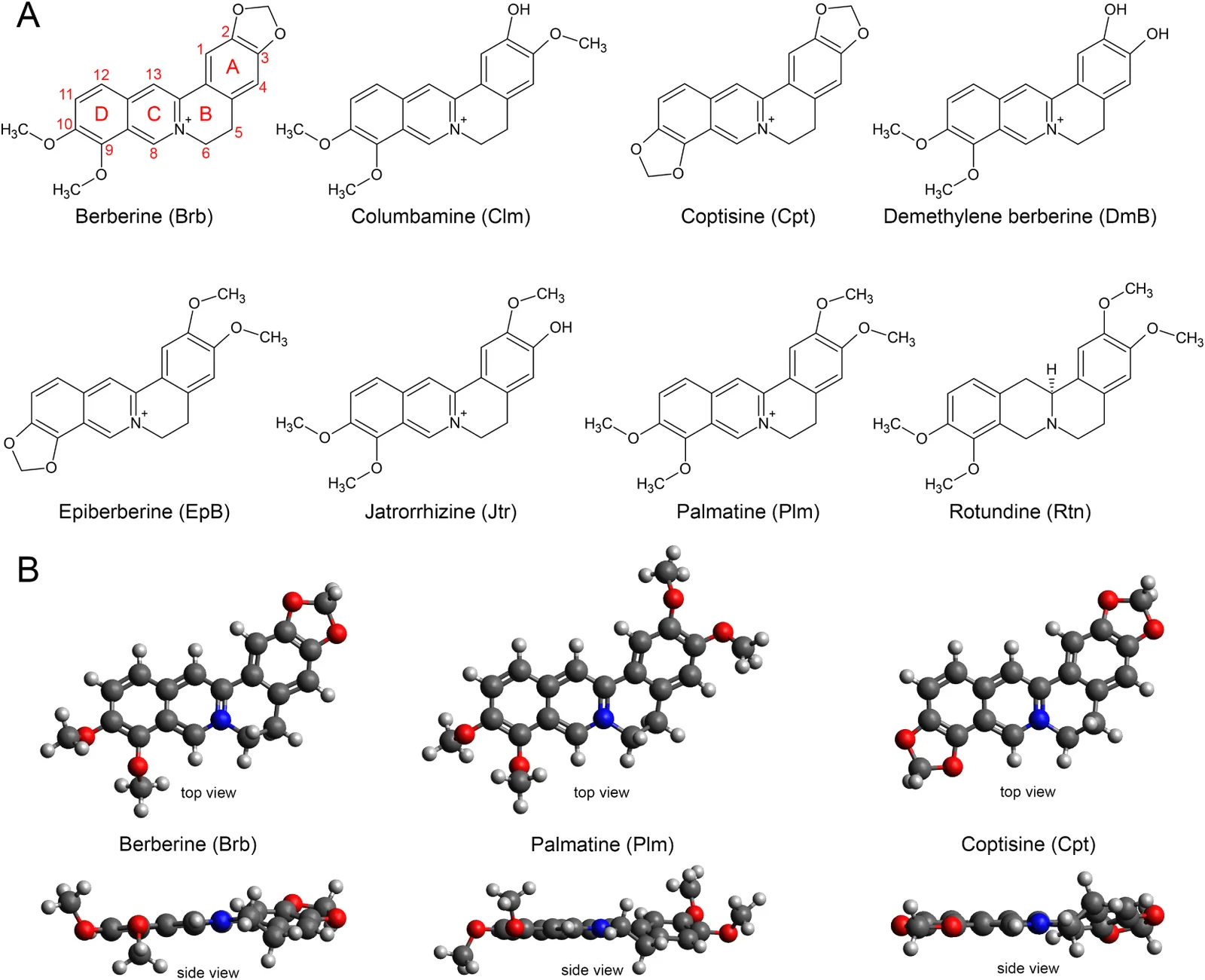
Structural diversity and planarity of protoberberine alkaloids. (A) Chemical structure of Brb, with atoms of the protoberberine core numbered according to Wang and co-workers,^39^ and of the indicated PBAs investigated in the present work. (B) Top and side views of Brb and the indicated PBA, highlighting the differences in molecular planarity among the compounds.

Recent investigations have also highlighted a specific protective mechanism based on the interaction of this compound with the cell membrane, inhibiting the binding of misfolded toxic protein oligomers.^7^ Indeed, it was recently demonstrated that Brb mitigates the neurotoxicity of misfolded protein oligomers without binding directly to the protein aggregates but rather by interacting with and crossing the lipid bilayer. Specifically, in liposomes used as models of the plasma membrane, Brb was shown to effectively inhibit the binding of toxic model HypF-N oligomers to the lipid surface, thereby preventing the subsequent disruption of bilayer integrity.^7^ This protective effect was further confirmed in human SH-SY5Y neuroblastoma cells, where Brb treatment significantly increased cell viability and reduced the damage induced by extracellular HypF-N and Aβ_42_ oligomers.^7^ Furthermore, it was observed that Brb crosses the plasma membrane to reach and interact with intracellular molecular targets, as previously reported.^7,11–14^ Ultimately, these results remarked that the efficacy of Brb is strictly dependent on its ability to both interact with the cell membrane to inhibit the action of misfolded oligomers^7^ and cross it to reach its intracellular molecular targets.^13–18^ The initial intercalation into the lipid bilayer is proposed as a critical event that may underlie both the direct protection of the membrane and the cellular uptake of the compound.

However, the therapeutic potential of Brb is hindered by its difficulty to intercalate and cross the intestinal epithelium and blood–brain barrier. Indeed, despite remarkable pharmacological activity, the clinical use of Brb is limited by low oral bioavailability (<1%) and modest blood–brain barrier permeability.^8,9,19^ This poor bioavailability arises from both membrane- and nonmembrane-related factors. Membrane barriers include poor intestinal absorption and active intestinal efflux transporters, whereas nonmembrane factors include self-aggregation in the stomach and extensive first-pass metabolism in the liver and gut, which further reduce systemic availability.^8,9,19^ Once absorbed, Brb rapidly distributes into tissues, accumulating in the liver, kidneys, heart, muscles, and pancreas, at concentrations far exceeding those in plasma.^19,20^ In neurodegenerative diseases, delivery to the brain is further restricted by the blood–brain barrier. Brb can cross the barrier to some extent and persist in specific brain regions, but the passage is far from optimal.^8^ Consequently, the interaction of Brb with lipid membranes is likely a critical factor governing its pharmacokinetics and biological effects.

The importance of studying how Brb interacts with and crosses lipid membranes carries therefore dual significance as it dictates both its pharmacological effects and systemic bioavailability. The specific structural features of Brb governing its interaction with the membrane and subsequent membrane passage remain, however, to be fully elucidated. It is well-established that parameters such as lipophilicity, molecular size, and number of hydrogen bond donors/acceptors heavily influence the passive membrane permeation of small molecules.^21–24^ Accordingly, alkylation and acylation of Brb at various positions improve bioavailability.^25^ However, in addition to these classical descriptors, other parameters have been put forward to determine passive diffusion of small molecules through the cell membrane. The identification of the key chemical groups or physicochemical parameters of a small molecule responsible for a given biological action is generally performed through structure–activity relationship (SAR) studies, which are typically conducted by varying chemically modifiable moieties or using an array of natural molecules of the same class when these are available.^26,27^

In the present study, we took advantage of the existence of various botanical compounds that are structurally very similar to Brb, often representing its metabolic intermediates or its further metabolic derivatization.^28,29^ Specifically, the SAR of a library of eight berberine analogues, namely, protoberberine alkaloids (PBAs), was explored, including columbamine (Clm), coptisine (Cpt), demethyleneberberine (DmB), epiberberine (EpB), jatrorrhizine (Jtr), palmatine (Plm), and rotundine (Rtn), alongside the parent compound Brb (Figure 1A). These specific analogues were selected not only for their amenability to isolation but also for their emerging roles as putative neuroprotective drugs; for instance, Cpt and Jtr have been shown to mitigate oxidative stress and apoptosis in AD and PD models,^30,31^ while DmB and Rtn exhibit enhanced bioavailability and significant central nervous system activity.^32,33^

The use of this library offers a methodological advantage, because it leverages naturally occurring compounds to explore systematic, subtle variations in benzylisoquinoline substituents and scaffold saturation, thereby bypassing the need for *de novo* chemical synthesis. Their natural origin can also correlate with a more favorable safety profile compared to that of purely synthetic compounds. SAR analysis was employed here to determine how the structure of Brb and, more generally, of the modifiable benzylisoquinoline scaffold governs its ability to interact with lipid membranes and interfere with misfolded protein oligomer binding and toxicity. By combining biophysical assays on large unilamellar vesicles (LUVs) with cellular assays on human neuroblastoma cells (SH-SY5Y), this study demonstrates that while Brb hydrophobicity and size are essential for membrane interaction, molecular planarity emerges as the strongest correlate for the membrane-mediated protection exerted by this class of compounds, with implications for both pharmacological activity and bioavailability.

## Results and Discussion

### Molecular Structures and Structural Determinants of Protoberberine Alkaloids

Natural berberine analogues, normally named PBAs, are a group of alkaloids sharing a 5,6-dihydrodibenzo­[a,g]­quinolizinium (C_17_H_14_N^+^) skeleton,^34–37^ and Brb is the most widely known compound among them (Figure 1A). This scaffold consists of four fused hexagonal rings, typically labeled as rings A, B, C, and D.^34^ Rings C and D form the isoquinoline moiety, while the partially saturated ring B (sharing the nitrogen atom with ring C) and ring A complete the protoberberine core. The diversity among the eight PBAs investigated in this work arises from the pattern of substituents (hydroxy, methoxy, or methylenedioxy groups) at positions 2 and 3 of ring A and positions 9 and 10 of ring D and the oxidation state of ring C (Figure 1A).

Table 1 lists the molecular weight (MW), number of hydrogen bond donors and acceptors (*N*_HB_), and hydrophobicity, expressed as the logarithm of the octanol/water partition coefficient (log­(*K*_ow_)), of the eight PBAs, as these parameters are known to affect membrane permeation.^21,22^ log­(*K*_ow_) values were derived from the experimental value of Brb (−1.5), which is the only derivative in the library to have been determined experimentally.^38^ The log­(*K*_ow_) values for the remaining seven PBAs were determined through chemical inference, calculating the contribution of specific substituents (e.g., hydroxy vs methoxy groups) to the parent scaffold’s partition coefficient, a standard predictive approach in medicinal chemistry. Table 1 also lists the deviation from the molecular planarity for the eight compounds, hypothesized here as a potential determinant of membrane interaction and permeation. This was quantified by counting the number of non-hydrogen atoms (O, C, N) bound covalently to other atoms that were not present in rings or sp^2^-hybridized, thus having the conformational freedom to deviate from the main molecular plane of the molecule. Brb displays a largely planar structure, with the methyl substituents at positions 9 and 10 protruding from the molecular plane and resulting, therefore, in two out-of-plane atoms (Table 1, Figure 1B). Small deviations from planarity are observed for several PBAs, including Clm, Jtr, DmB, EpB, and Brb itself, which present two to three out-of-plane atoms (Table 1). By contrast, Cpt retains a highly planar scaffold, with zero out-of-plane atoms (Table 1, Figure 1B), whereas Plm and Rtn exhibit a more pronounced three-dimensional character, with four and five out-of-plane atoms, respectively (Table 1, Figure 1B). In the case of Rtn, this is due to the loss of the double bond on the nitrogen of ring C (Figure 1A).

**Table 1:** Molecular Weight, Hydrophobicity, Number of H-bonds Donor/Acceptors and Molecular Planarity of the PBAs Investigated in This Work

PBA compound	MW (g/mol)^a^	Hydrophobicity (log(*K*_ow_))^b^	Number of H-bonds (*N*_HB_)^c^	Out-of-plane atoms^d^
Berberine (Brb)	336.4	–1.50	4	2
Columbamine (Clm)	338.4	–1.90	5	3
Coptisine (Cpt)	320.3	–1.66	4	0
Demethyleneberberine (DmB)	324.3	–2.49	6	2
Epiberberine (EpB)	336.4	–1.50	4	2
Jatrorrhizine (Jtr)	338.4	–1.90	5	3
Palmatine (Plm)	352.4	–1.34	4	4
Rotundine (Rtn)	355.4	–1.34	5	5

a Molecular weight, expressed in g/mol.

b Hydrophobicity, expressed as the logarithm of the octanol/water partition coefficient (log *K*_ow_), derived from the experimental value of −1.5 of Brb,^38^ through chemical inference.

c Sum of the number of H-bond acceptors and donors.

d Deviation from the molecular planarity quantified by the number of non-hydrogen atoms (O, C, N) bound covalently to other atoms that were not present in cycles or hybridized sp^2^.

These structural differences provide a framework to investigate whether variations in MW, hydrophobicity, *N*_HB_, and molecular planarity influence the interaction of PBAs with lipid membranes.

### Structure-Dependent Affinity of Protoberberine Alkaloids for LUV Membranes

The affinity of PBAs for lipid membranes was first investigated using fluorescence quenching experiments on TMA-DPH-labeled LUVs. Given the absence of biological molecular targets in the LUV lumen, Brb was previously shown to remain within the membrane in this system, as a result of a membrane/buffer partition coefficient (log *P*) of 3.17 ± 0.11.^7^ TMA-DPH is a fluorescent probe which incorporates within the polar head region of the bilayer and reports on molecular interactions occurring at the outer portion of the membrane.^40–42^ Incubation of TMA-DPH-labeled LUVs with increasing concentrations of Brb resulted in a concentration-dependent quenching of fluorescence emission at 430 nm (Figure 2A), as previously observed.^7^ A similar quenching behavior was observed for most PBAs, with the exception of Rtn, indicating a negligible interaction of this compound with the LUV lipid membrane (Figure 2A).

**Figure 2:**
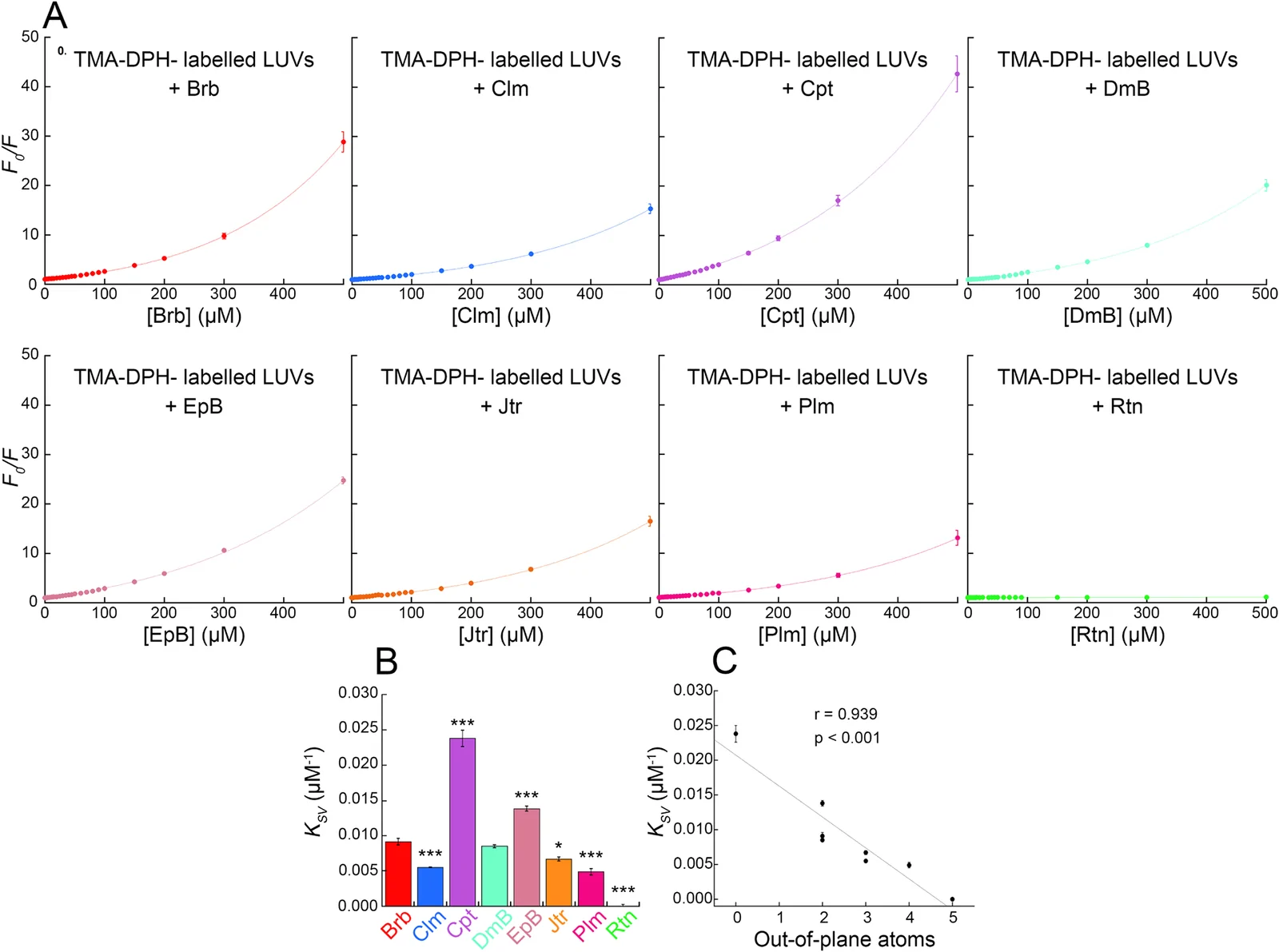
Molecular planarity enhances membrane affinity of protoberberine alkaloids. (A) Stern–Volmer plots of TMA-DPH-labeled LUVs incubated with increasing concentration of the indicated PBA. Fluorescence data on the *y*-axis are reported as the ratio of fluorescence in the absence (*F*_0_) and presence (*F*) of PBAs at 430 nm. (B) *K*_SV_ values, representing the affinity constants between the PBAs and the membrane of LUVs, were obtained by fitting the *F*_0_/*F* at 430 nm versus [PBA] with eq 1, as described in the Experimental Section. Data are expressed as mean ± SEM of 3–6 experiments. * and *** indicate *p* values <0.05 and <0.001, respectively, relative to *K*_SV_ of Brb (statistical analysis: ANOVA test followed by the Bonferroni post hoc test). (C) Correlation between *K*_SV_ and the number of out-of-plane atoms of the PBAs.

Stern–Volmer analysis revealed significant differences in affinity for the LUV membrane among the analogues, as quantified by the dynamic Stern–Volmer constant (*K*_SV_) values obtained from fitting the fluorescence data with an extended Stern–Volmer model, which reflects the efficiency of fluorescence quenching as a function of quencher concentration and thus the ability of each compound to access and interact with the membrane region surrounding the probe (Figure 2B). Rtn has a negligible *K*_SV_ value approaching zero. Clm, Jtr, and Plm displayed significantly lower *K*_SV_ values compared to Brb, indicating a reduced affinity for the membrane. By contrast, Cpt and EpB showed significantly higher *K*_SV_ values, whereas DmB exhibited a *K*_SV_ value comparable to that of the parent compound.

When the *K*_SV_ values were plotted as a function of MW, log­(*K*_ow_), or *N*_HB_, no correlations were observed, with *p* > 0.05 (Figure S1A,B,C). This does not imply that these factors are not relevant because they are well-known to affect membrane binding and permeation.^21–24^ The absence of correlations rather stems from the high structural similarity among the eight compounds studied here and the very narrow distributions of values for these three parameters across the eight compounds. In other words, the minimal changes in these three determinants did not explain the experimentally determined differences in *K*_SV_ values. By contrast, when the *K*_SV_ values were plotted as a function of the number of out-of-plane atoms, a clear negative correlation emerged (Figure 2C), indicating that deviation from molecular planarity is associated with a decrease in membrane affinity.

### Structure-Dependent Interaction Kinetics of Protoberberine Alkaloids with LUV Membranes

To investigate whether structural differences among PBAs also affected the kinetics of membrane interaction beyond the interfacial region, time-dependent fluorescence quenching experiments were performed on DPH-labeled LUVs. Unlike TMA-DPH, which preferentially resides near the water–membrane interface, DPH partitions deeply into the hydrophobic core of lipid bilayers and reports on molecular events occurring within the membrane interior.^40,41,43,44^ DPH was specifically selected for these kinetic experiments to monitor the subsequent diffusion and penetration of the molecules into the hydrophobic core of the bilayer with the potential to cross it in cells.

Upon incubation with 30 μM PBA, DPH-labeled LUVs displayed time-dependent fluorescence quenching over the 0–4000 s time window (Figure 3A). The experimental time courses were well-described for all PBAs by a double exponential function (eq 3), accounting for a rapid quenching component (*k*_fast_), occurring within seconds, and a slower component (*k*_slow_) developing over minutes. With the exception of Rtn, which showed a dramatically decreased *k*_fast_ value, the *k*_fast_ values of the seven remaining PBAs were similar, with differences spanning only a factor of 1.8. This indicates that the fast phase is likely to be associated with a nonspecific interaction that does not depend significantly on the molecular structure of the PBAs. By contrast, the distribution of *k*_*slow*_ values was wider, revealing differences in interaction kinetics (Figure 3B). In particular, Cpt displayed a significantly higher *k*_slow_ value compared to Brb, indicating faster slow-phase interaction kinetics with the lipid membrane. EpB also showed a slightly increased *k*_slow_ value relative to Brb. Rtn displayed a dramatically lower *k*_slow_ value, which was not measurable within our time window, showing therefore an upper bound of 10^–5^ ± 10^–4^ s^–1^. All other analogues exhibited *k*_slow_ values comparable to or slightly lower than that of Brb.

**Figure 3:**
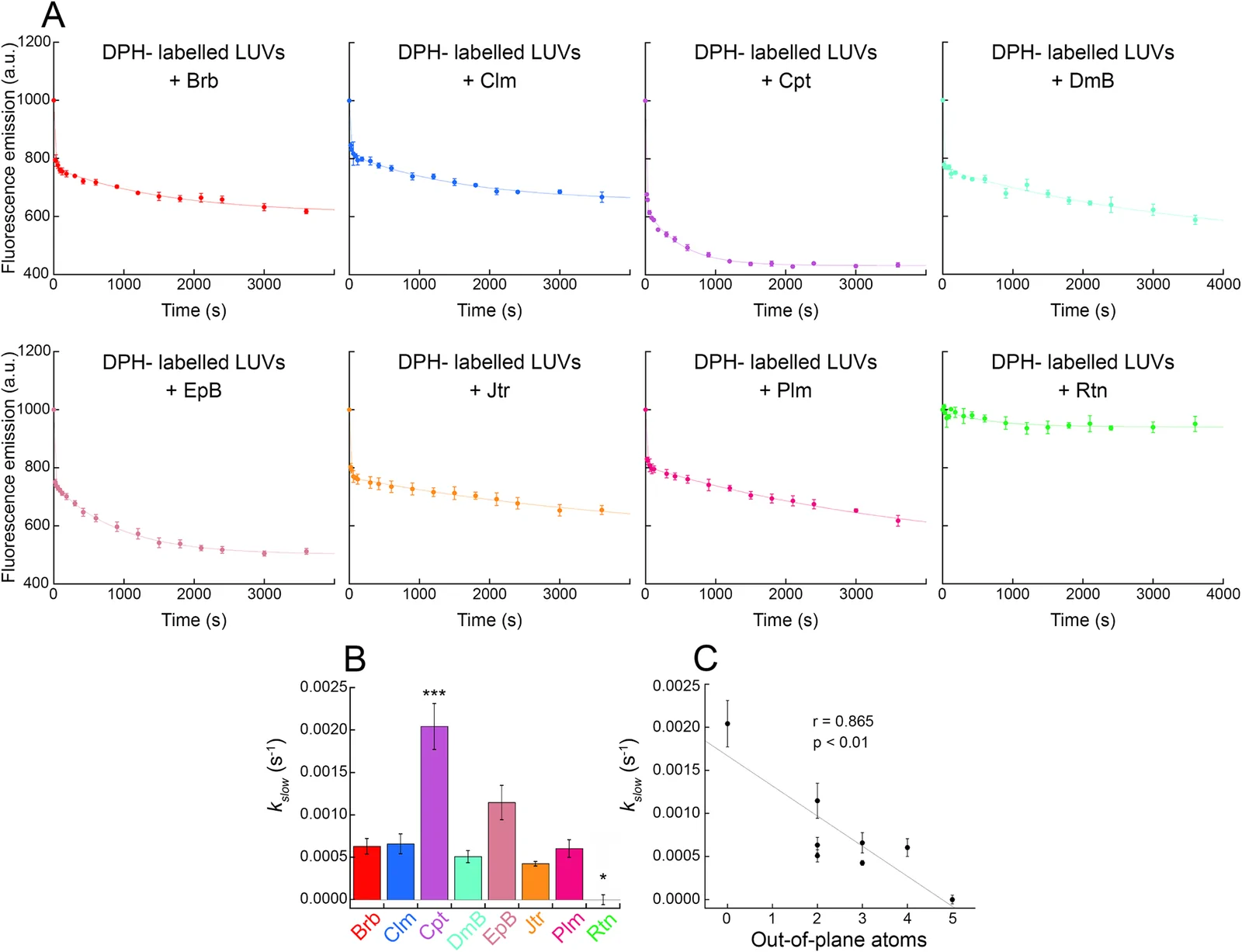
Molecular planarity accelerates membrane interaction kinetics of protoberberine alkaloids. (A) Fluorescence emission at 430 nm of DPH-labeled LUVs incubated with 30 μM of the indicated PBA over time. Data points were fitted with a double exponential function (eq 3). (B) *k*_slow_ values, representing the slower quenching component occurring over minutes, were obtained by fitting the plots with eq 3, as described in the Experimental Section. Data are expressed as mean ± SEM of 3–5 experiments. *** indicates *p* value <0.001, relative to *k*_slow_ of Brb (statistical analysis: ANOVA test followed by the Bonferroni post hoc test). (C) Correlation between *k*_slow_ and the number of out-of-plane atoms of the PBAs.

When *k*_slow_ values were plotted as a function of the number of out-of-plane atoms, a clear inverse correlation was observed (Figure 3C), consistent with the relationship previously identified for affinity. This implies that compounds characterized by higher planarity exhibited higher membrane affinity and also faster interaction kinetics. Similarly to the analysis performed for *K*_SV_, correlations were not observed (*p* > 0.05) when the *k*_slow_ values were plotted *versus* MW, log­(*K*_ow_), or *N*_HB_ (Figure S1D,E,F).

### Structure-Dependent Effects of Protoberberine Alkaloids on Membrane Dynamics

The effects of PBAs on membrane dynamics were investigated by fluorescence anisotropy measurements on TMA-DPH-labeled LUVs. Fluorescence anisotropy of TMA-DPH embedded in LUVs reports on the rotational mobility of the probe within the interfacial region of the lipid bilayer, providing information on membrane order and dynamics.^41,44–46^ Incubation of TMA-DPH-labeled LUVs with increasing concentrations of PBAs resulted in a concentration-dependent increase in fluorescence anisotropy (Figure 4A), indicating a reduction in probe rotational mobility and an increase in membrane order.

**Figure 4:**
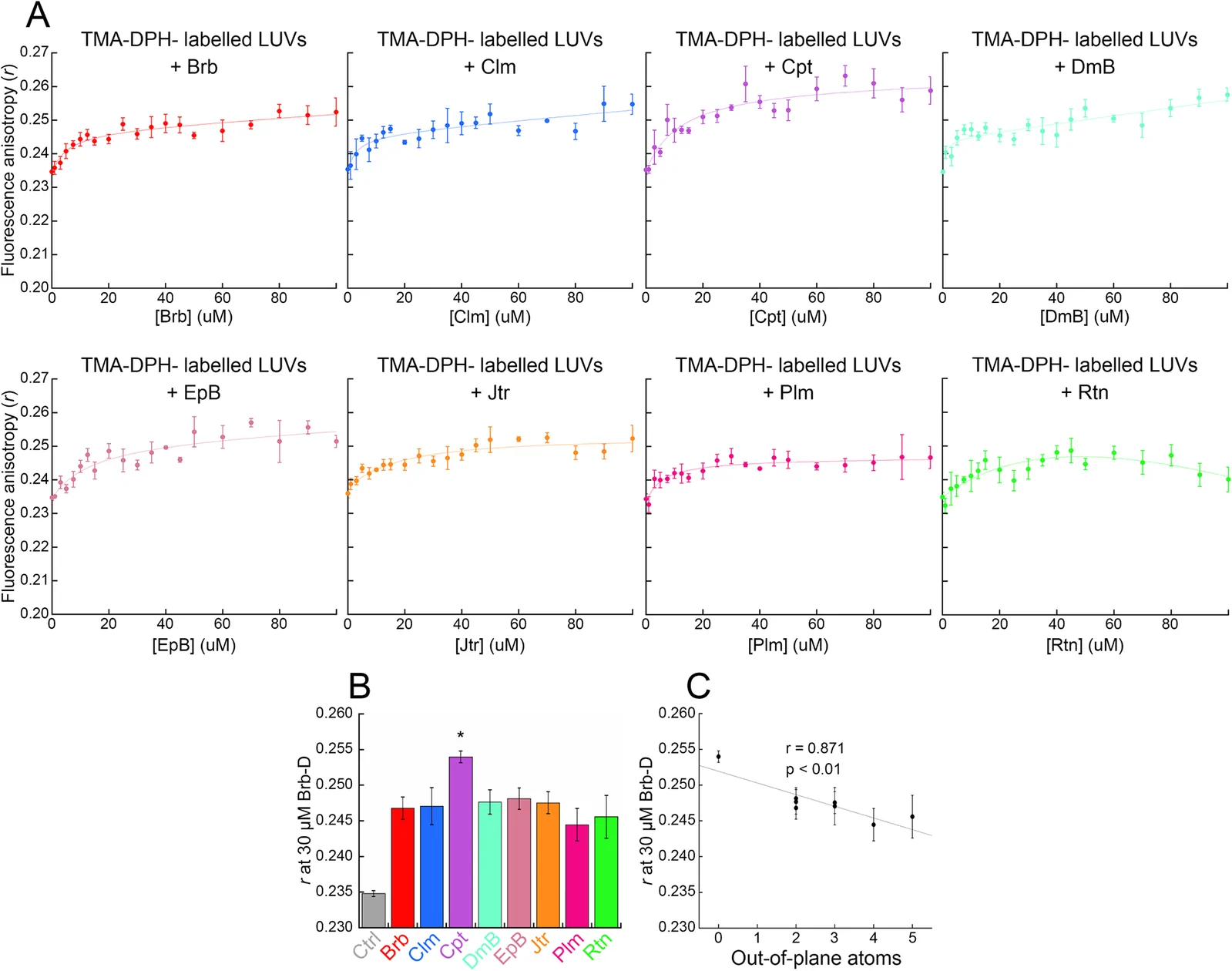
Protoberberine alkaloids increase membrane order in a planarity-dependent manner. (A) Fluorescence anisotropy (*r*) at 430 nm of TMA-DPH-labeled LUVs incubated with increasing concentrations of the indicated PBA and fitted with eq 2. (B) *r* values in the absence (ctrl) and presence of 30 μM of PBAs. Data are expressed as mean ± SEM of 3–6 experiments. * indicates P value <0.05, relative to *r* at 30 μM of Brb (statistical analysis: unpaired *t*-test). (C) Correlation between *r* values at 30 μM PBAs and the number of out-of-plane atoms.

Comparison of anisotropy values at a fixed concentration (30 μM) of PBAs revealed subtle differences among the analogues (Figure 4B). Cpt induced the largest increase in anisotropy, consistent with a pronounced increase in membrane order. By contrast, Plm and Rtn produced a less marked increase in anisotropy, although still significantly higher than control conditions. The remaining PBAs displayed intermediate effects, with anisotropy values comparable to or moderately higher than the value observed for Brb.

Despite the subtle and often nonsignificant differences observed, when anisotropy values measured at 30 μM (*r* at 30 μM) were plotted as a function of the number of out-of-plane atoms, a clear and statistically significant inverse correlation was observed (Figure 4C), indicating that compounds characterized by a higher planarity induced higher TMA-DPH anisotropy values. Statistically significant inverse correlations were also obtained when considering *r* values at other PBA concentrations, such as 20, 50, or 100 μM. This correlation mirrors the relationships previously observed for membrane affinity and interaction kinetics. Again, significant correlations were not observed (*p* > 0.05) between TMA-DPH *r* values at 30 μM PBA and either MW, log­(*K*_ow_), or *N*_HB_ (Figure S1G,H,I).

### Structure-Dependent Binding Inhibition of HypF-N Toxic Oligomers to LUV Membranes by Protoberberine Alkaloids

We then investigated the ability of PBAs to inhibit the binding of toxic HypF-N oligomers of type A (OAs) to lipid membranes of LUVs. HypF-N OAs were selected because they were found to have toxic effects similar to those of Aβ_42_ oligomers with different tests^47,48^ and because a specific assay has been previously set up for Brb and other compounds.^7,49^ We monitored the OA-induced fluorescence quenching of TMA-DPH embedded in LUVs by various OA concentrations at a fixed (30 μM) concentration of PBA. Under control conditions without PBAs, incubation of TMA-DPH-labeled LUVs with increasing concentrations of OAs resulted in a linear increase of the *F*_0_/*F* ratio, consistent with concentration-dependent quenching of the probe (Figure 5A), as previously reported.^7,49^ Preincubation of LUVs with Brb markedly altered the OA-induced quenching profile. In line with previous observations, Brb caused a pronounced flattening of the *F*_*0*_/*F* curve at low OA concentrations, indicating a strong reduction or absence of OA-induced quenching. At higher OA concentrations, quenching was still observed; however, *F*_0_/*F* values remained significantly lower than those measured in the absence of PBAs, suggesting a partial but persistent displacement of OAs from the membrane.^7^ A similar behavior was observed in the presence of Cpt and EpB. Notably, Cpt produced a more pronounced effect than that of Brb, whereas EpB displayed a displacement profile closely resembling that of the parent compound. By contrast, preincubation with Clm, DmB, Jtr, Plm, or Rtn produced only minor effects on OA-induced quenching, with *F*_*0*_ */F versus* OA concentration curves largely overlapping, in some cases, with those obtained in control LUVs without PBAs (Figure 5A).

**Figure 5:**
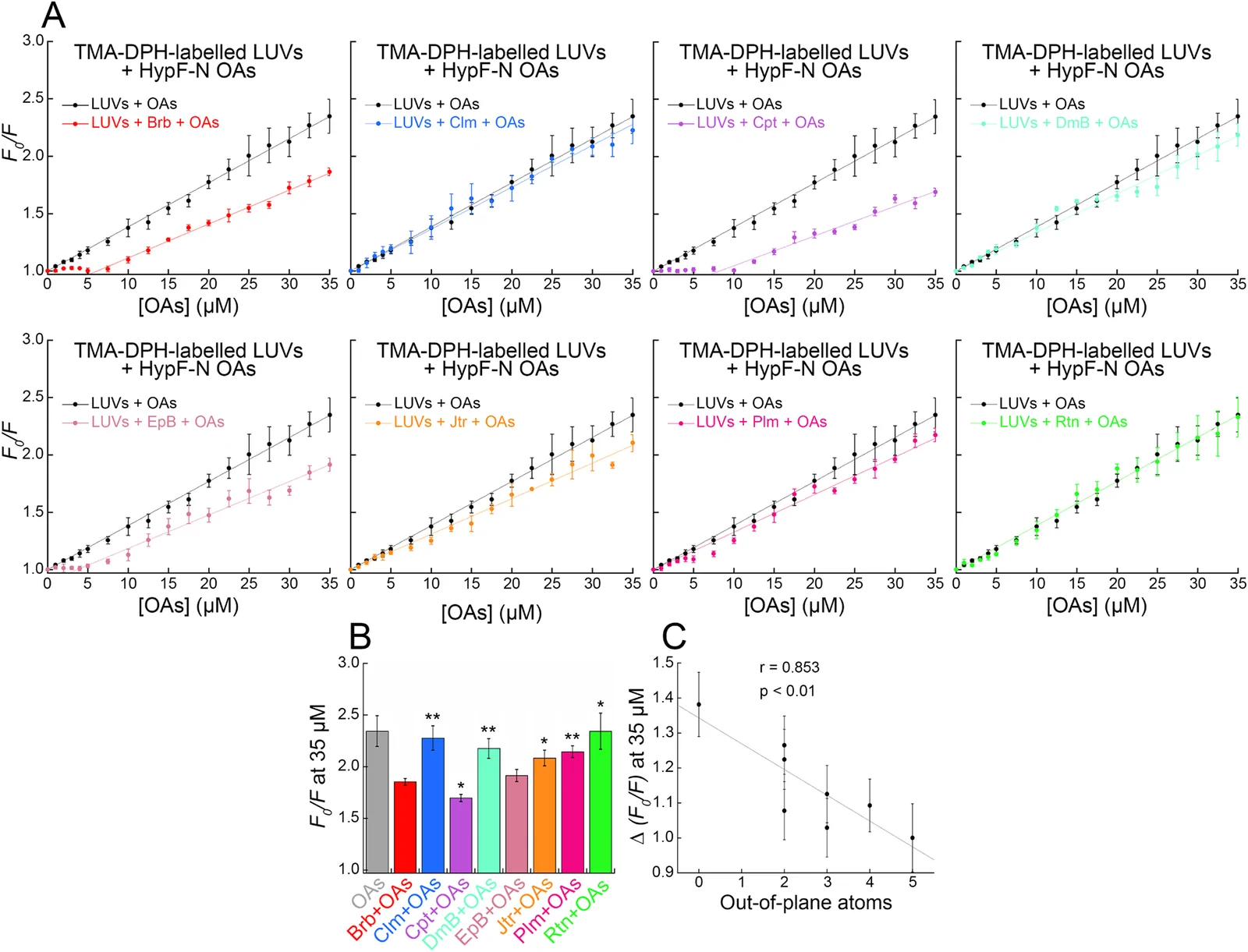
Protoberberine alkaloids displace HypF-N oligomers from membranes in a planarity-dependent manner. (A) Stern–Volmer plots reporting the ratio of fluorescence of TMA-DPH-labeled LUVs in the absence (*F*_0_) and presence (*F*) of various concentrations of toxic HypF-N oligomers of type A (OAs), in the absence (black) and presence of the indicated PBA (30 μM) preincubated with LUVs. The straight lines through the data points represent the best fits to eq 4 in the absence of PBAs and in LUVs preincubated with 30 μM Clm, DmB, Jtr, Plm, and Rtn and to eq 5 in LUVs preincubated with Brb, Cpt, and EpB. Data are expressed as mean ± SEM of 4 experiments. (B) *F*_0_/*F* values at 35 μM OAs obtained from fitting the data with eqs 4 and 5 in the absence (gray) and presence (color) of the indicated PBA. * and ** indicate *p* values <0.05 and <0.01, respectively, relative to *F*_0_/*F* values at 35 μM OAs in the presence of Brb (statistical analysis: unpaired *t*-test). (C) Correlation between the ratio of *F* *_0_*/*F* values at 35 μM OAs in the absence and presence of PBAs (Δ­(*F*_0_/*F*)) and the number of out-of-plane atoms of each compound.

Quantitative comparison of *F*_0_/*F* values at 35 μM OAs confirmed these observations (Figure 5B). When the reduction in OA-induced quenching (Δ­(*F*_0_/*F*)) was plotted as a function of the number of out-of-plane atoms, a clear inverse correlation was observed (Figure 5C), indicating that compounds with a higher degree of planar structure display a higher ability to displace OAs from the membrane. Similarly to all other analyses reported in the previous sections, Δ­(*F*_0_/*F*) was not found to correlate with MW, log­(*K*_ow_), or *N*_HB_ (Figure S1J,K,L).

Since all the parameters analyzed so far on LUVs—namely, *K*_SV_, *k*_slow_, *r,* and Δ­(*F*_*0*_/*F*)—correlate negatively with the number of out-of-plane atoms, statistically significant correlations were also found between all pairs of these four parameters (Figure S2A–F). This shows that the more planar a PBA, the higher its affinity for the LUV membrane, the more rapid its rate of binding, the more rigid the resulting membrane, and the more resistant the membrane appears to the binding of toxic misfolded protein oligomers.

### Protoberberine Alkaloids Do Not Markedly Alter the Secondary Structure or Hydrophobic Exposure of HypF-N Oligomers

In principle, the displacement of HypF-N OAs from LUVs induced by PBAs could arise from either a direct binding of PBAs to the LUV membrane that changes its physicochemical properties to a degree sufficient to prevent OA-LUV binding or from the direct binding of PBAs to OAs, preventing the binding of the resulting complex with the LUV membrane. To assess the second possibility, we investigated the impact of these compounds on the secondary structure and surface hydrophobicity of OAs using far-UV circular dichroism (CD) spectroscopy and ANS fluorescence.

OAs were formed in the absence or presence of increasing molar ratios of PBAs (1:0.25, 1:0.5, 1:1) and their CD spectra were recorded at the final conditions of 10 μM OAs (monomer equivalents) and 2.5–10.0 μM PBAs (Figure 6A). In the absence of PBAs, the CD spectrum was predominantly β-sheet with contributions of α-helix and disordered structure, as expected for these oligomeric species.^50,51^ No major differences in the overall shape or position of the CD spectral minima were observed in the presence of PBAs, indicating that the secondary structure content of the oligomers was largely preserved upon incubation with the compounds. A modest flattening of the CD spectra was detected in several cases, particularly at higher compound-to-protein ratios; however, this effect was not associated with qualitative changes in spectral features and is consistent with a reduction in high tension (HT) voltage caused by the compound rather than a substantial structural rearrangement of the oligomers, as previously reported for compound–oligomer interactions,^7,52,53^ including Brb-OAs.^7^

**Figure 6:**
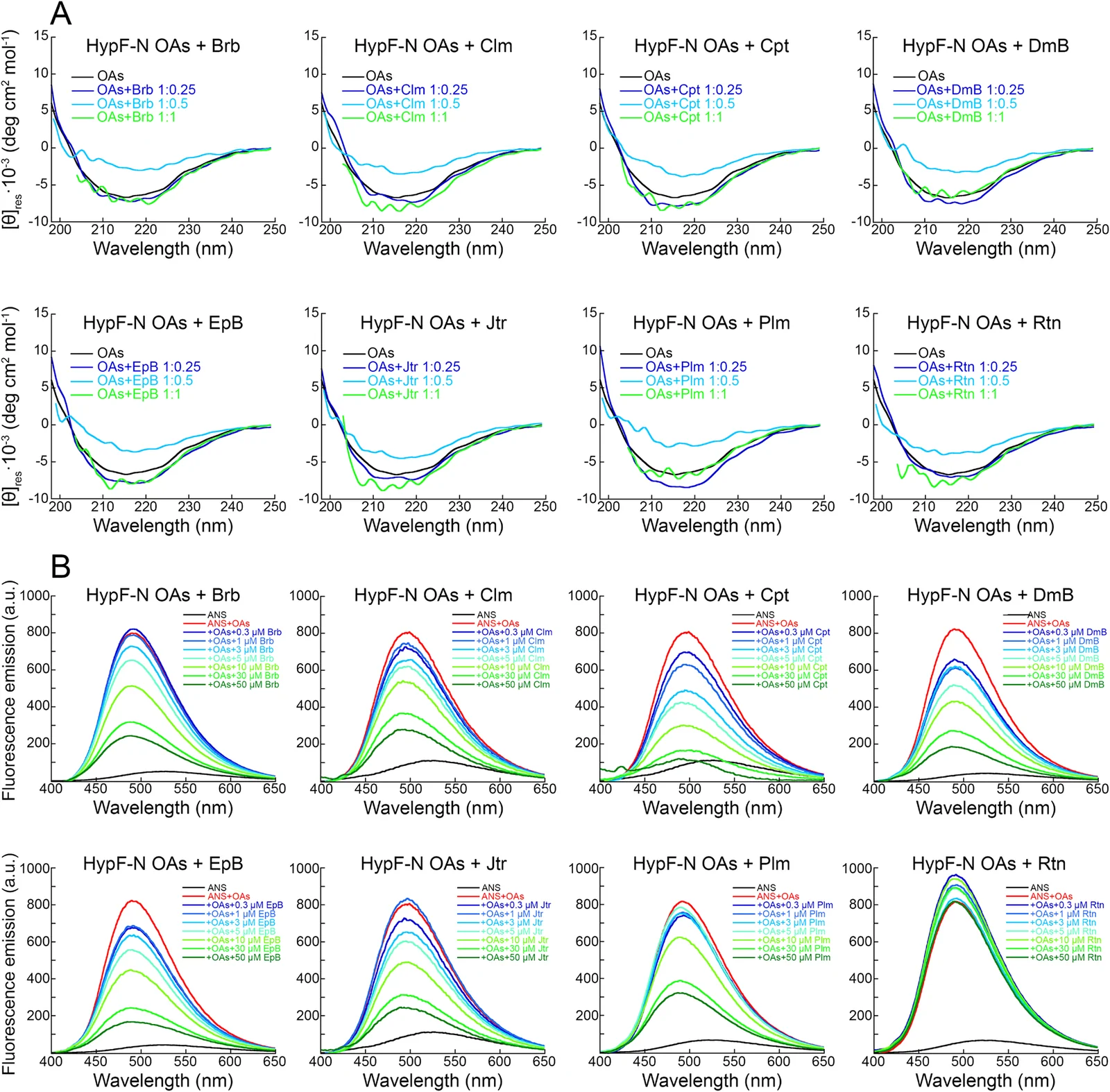
Protoberberine alkaloids do not substantially remodel HypF-N oligomers. (A) Far-UV CD spectra of 10 μM HypF-N OAs (monomer equivalents) oligomerized in the absence and presence of different concentrations (2.5–10.0 μM) of the indicated PBA. (B) Fluorescence spectra of 100 μM ANS with 10 μM HypF-N OAs (monomer equivalents) incubated with increasing concentration (0.3–50 μM) of the indicated PBA.

The exposure of hydrophobic regions on the surface of OAs was evaluated by ANS fluorescence (Figure 6B). In the presence of OAs alone (10 μM monomer equivalents), ANS (100 μM) displayed a strong fluorescence signal, consistent with binding to exposed hydrophobic patches. The addition of increasing concentrations of PBAs from 0.3 to 50 μM (with the exception of Rtn) resulted in a concentration-dependent decrease in ANS fluorescence intensity. Importantly, this decrease was not accompanied by a blue-to-red spectral shift characteristic of ANS release into the aqueous environment, indicating that the compounds reduce ANS fluorescence without fully abolishing hydrophobic exposure or displacing the dye from the oligomer surface. Control experiments confirmed previously that Brb did not directly interact with ANS in the absence of OAs.^7^

Taken together, these data indicate that PBAs do not induce major alterations in the secondary structure or hydrophobic surface exposure of HypF-N toxic oligomers. Therefore, the displacement of oligomers from LUV membranes observed in the presence of selected compounds argues against direct disaggregation or remodeling of the oligomers and instead supports a membrane-mediated mechanism.

### Protoberberine Alkaloids Reduce Oligomer-Induced Calcium Influx in Cells

After assessing the ability of the various PBAs to bind to the membrane of reconstituted liposomes and displace toxic oligomers from it, all with a degree correlating positively with their molecular planarity, we aimed at assessing whether the various PBAs were also protective to toxic misfolded protein oligomers interacting with the membrane in cell cultures. To this aim, we used a cell model to mimic the membrane damage mediated by toxic misfolded protein oligomers based on undifferentiated neuroblastoma SH-SY5Y cells and extracellularly added Aβ_42_ amyloid-derived diffusible ligands (ADDLs) as a source of toxic misfolded protein oligomers. Aβ_42_ ADDLs were used for their wide use, reproducibility in formation, and possibility of checking their formation with appropriate quality control experiments and because they were also found in AD brains, unlike age-matched control brains.^54–56^ We measured the increase in intracellular Ca^2+^ levels induced by the interaction of ADDLs with the cell membrane. This response represents one of the earliest biochemical alterations following oligomer–membrane interaction.^57–59^ Specifically, oligomers promote Ca^2+^ influx by destabilizing the plasma membrane and by activating NMDA and AMPA receptors.^57,60,61^

Undifferentiated SH-SY5Y cells were first treated with PBAs to rule out any intrinsic toxic effects of the compounds. After a 20 min treatment with a concentration of 10 μM of any of the tested PBAs, no significant increases in intracellular Ca^2+^ levels were observed by confocal scanning microscopy (Figure S3A). Similarly, no significant decreases in cell viability were detected following a 24 h treatment, as evaluated by the 3-(4,5-dimethylthiazol-2-yl)-2,5-diphenyltetrazolium bromide (MTT) assay (Figure S3B). SH-SY5Y cells were then treated with PBAs (from 0.0009 to 30 μM) for 10 min, after which toxic Aβ_42_ ADDLs were added to the culture medium for another 10 min at a concentration of 0.5 μM (monomer equivalents). The intracellular Ca^2+^ levels were then measured immediately with scanning confocal microscopy for all conditions of treatment and converted into a normalized response (*R*) for all eight PBAs, as described in the Experimental Section (eq 7). (Figure 7A). Plots of *R versus* PBA concentration were analyzed with the Hill equation to determine the protective efficacy of the various compounds (Figure 7B), as exemplified by the values of maximum protection (*E*_max_) and of PBA concentration resulting in half protection (*EC*_50_). Brb was found to have an *EC*_50_ value of 0.149 ± 0.064 μM and an *E*_max_ value of 100.6 ± 7.3% (Figure 7B). *E*_max_ values showed a distribution of values from *ca*. 55% to 100% across the eight PBAs (Figure 7B,C). *EC*_50_ values also showed considerable variability, ranging from the lowest value (highest protection) exhibited by Cpt, which was *ca*. 4-fold lower than that of Brb, to the highest (lowest protection) featured by Jtr, which was *ca*. 5.5-fold higher than that of Brb (Figure 7B,D).

**Figure 7:**
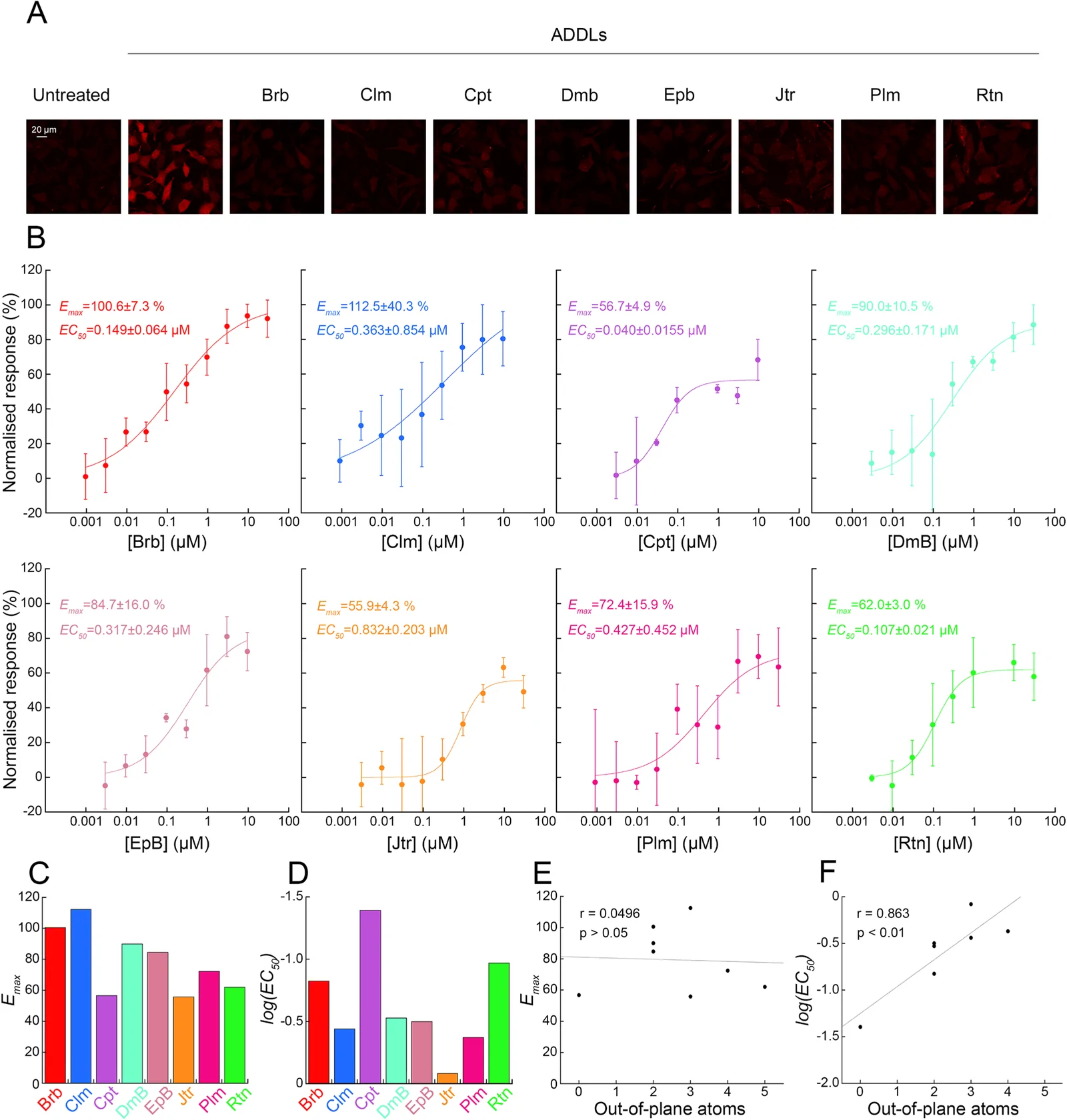
Protoberberine alkaloids protect cells from oligomer-induced Ca^2+^ dysregulation. (A) Representative confocal scanning microscopy images of free Ca^2+^ levels in SH-SY5Y cells pretreated or left untreated with the indicated PBA (10 μM, 10 min) and then exposed to 0.5 μM ADDLs (monomer equivalents) for another 10 min, where indicated. Cells were then loaded with the X-Rhod-1 AM red fluorescent probe. (B) Normalized dose–response curves obtained from Ca^2+^-derived fluorescence in SH-SY5Y cells treated as in (A), using increasing concentrations of PBAs and eq 7. Data are expressed as mean ± SEM of 3 experiments. (C,D) *E*_max_ and log­(*EC*_50_) values obtained by fitting the normalized dose–response curves with eq 8. (E,F) Plots of *E*_max_ and log­(*EC*_50_) values versus the number of out-of-plane atoms for PBAs.

While no correlation was found between *E*_max_ and the number of out-of-plane atoms (Figure 7E), a statistically significant correlation was found between *EC*_50_ and the same parameter, provided Rtn is excluded from the analysis (Figure 7F). These results can be rationalized by considering that Brb is known to pass through the membrane by a process of passive transport, either simple or facilitated diffusion,^62–66^ and to have different molecular targets inside the cell.^13–18^ Its protective action, therefore, is likely to correlate with the thermodynamics and kinetics of membrane binding and kinetics of crossing but also with the affinity for and effect on the various molecular targets. For this reason, *E*_max_ values were not found to correlate with the molecular planarity of PBAs, given the multiplicity of biological parameters involved when PBA concentrations are high. Nevertheless, a correlation was found when *EC*_50_ was taken into consideration in the analysis, as this reflects more closely the effects of the various PBAs on the compound–membrane binding and compound-induced membrane protection.

*EC*_50_ was not found to correlate with MW, log­(*K*_ow_), or *N*_HB_ (Figure S1M,N,O), whereas it was found to correlate with the *K*_SV_, *k*_slow_, *r,* and Δ­(*F*_0_/*F*) parameters determined on LUVs (Figure S2G–J). This reinforces the idea that the efficiency of a PBA in protecting cells from the toxic action of extracellular Aβ_42_ oligomers, probed as an influx of Ca^2+^ ions from the extracellular space into the cytosol, results, among other factors, from the thermodynamics and kinetics of its binding to the lipid bilayer of the membrane, thereby shielding the membrane from toxic protein oligomers and more effectively reaching the intracellular molecular targets. These effects are consistent with molecular planarity being an important determinant of membrane partitioning within this closely related series of compounds.

## Conclusions

The interaction between Brb and the cell membrane is critical, as it dictates both the intracellular bioavailability of the compound and its inherent ability to prevent the cytotoxic effects of misfolded protein oligomers, which play such an important role in the pathogenesis of neurodegenerative diseases. A comparison of protein-free model membranes and living cell systems allowed us to distinguish between membrane shielding and intracellular mechanisms underlying the protective activity of PBAs. In protein-free LUVs, the protective effect is entirely membrane-mediated, as more planar compounds partition more favorably, stiffen the bilayer, and displace toxic oligomers more effectively (Figure 8A). Accordingly, the potency of cellular protection (*EC*_50_) correlates with planarity-dependent membrane-binding parameters, whereas the maximal effect (*E*_max_) does not, likely reflecting the contribution of intracellular processes at higher compound concentrations (Figure 8A).

**Figure 8:**
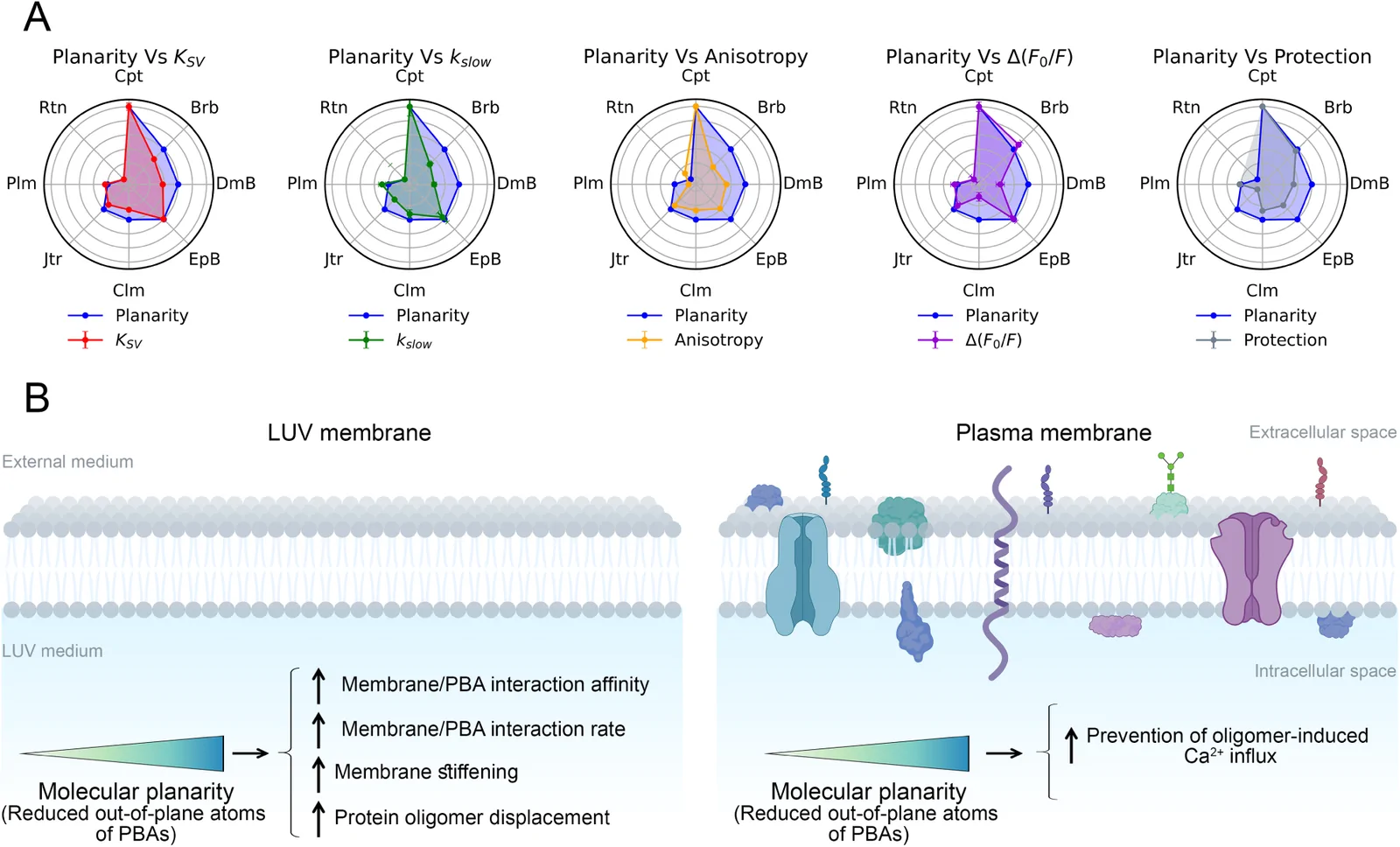
A planarity-dependent mechanism of membrane protection by protoberberine alkaloids. (A) Dual-axis radar charts comparing molecular planarity (blue, common axis across all five charts) with each of the five experimental readouts: *K*_SV_ (membrane affinity), *k*_slow_ (membrane interaction kinetics), TMA-DPH fluorescence anisotropy at 30 μM PBA (membrane order), Δ­(*F_0_*/*F*) at 35 μM HypF-N OAs (oligomer displacement), and protection (cellular activity). All values were min–max normalized across the eight compounds, with 1 representing the strongest effect and 0 the weakest. Planarity was scored as 1/(*n* + 1), where *n* is the number of non-hydrogen out-of-plane atoms (Table 1), to accommodate Cpt (*n* = 0). Protection was defined as log­(1/*EC*_50_), so that higher values denote greater potency. The visual coincidence between the planarity polygon and each parameter polygon reflects the pairwise correlations quantified in Figures 2C, 3C, 4C, 5C, and 7F. (B) Schematic summarizing the proposed mechanism. In protein-free LUVs (left), increasing molecular planarity of PBAs (i.e., fewer non-hydrogen out-of-plane atoms) is associated with stronger membrane binding affinity, faster membrane interaction kinetics, increased bilayer ordering, and more effective displacement of toxic HypF-N oligomers from the lipid surface. In SH-SY5Y cells (right), the same gradient of planarity is associated with greater protection against Aβ_42_ ADDL-induced Ca^2+^ influx, consistent with a primarily membrane-mediated mechanism of action. Figure created with BioRender.com.

Brb and its analogues comply with Lipinski’s rule of five and it is known that Brb can indeed interact with and pass through the cell membrane. We found, however, that the subtle variations in the physicochemical descriptors of Lipinski’s rule of five across the series, such as MW, log­(*K*_ow_), or *N*_HB_, do not account for the observed differences in membrane–molecule interaction kinetics and thermodynamics, most likely because the physicochemical changes are minimal across this group of compounds. We rather identify molecular planarity, quantified by the inverse of the number of non-hydrogen out-of-plane atoms, as the structural feature that discriminates membrane interaction efficacy within this narrow physicochemical series (Figure 8B). Analogues with higher planarity were found to partition more effectively, energetically, and kinetically, into the lipid bilayer, to induce a specific increase in membrane order, and to inhibit the membrane–oligomer interaction, as consistently revealed by fluorescence quenching and anisotropy experiments (Figure 8B). The same structural feature is likely to favor membrane permeation, enabling more rapid access to intracellular targets. Conversely, deviations from planarity were associated with reduced membrane affinity and markedly lower protective activity. Accordingly, Cpt and Rtn display the strongest and weakest membrane interactions, as they are the most and least planar compounds in the series, with zero and five non-hydrogen out-of-plane atoms, respectively. Brb also shows substantial membrane interaction, as it contains only two non-hydrogen out-of-plane atoms, although it is less planar than Cpt. The data support a mechanism in which protection is primarily mediated by modulation of the lipid bilayer rather than by major remodeling of protein oligomers.

In the context of drug discovery and medicinal chemistry, planarity is generally considered an undesirable feature because it is often associated with poor physicochemical properties and poor clinical success.^67,68^ Analyses of drug development pipelines have shown that compounds progressing toward approved drugs tend to exhibit higher carbon saturation and three-dimensional complexity, which can improve receptor complementarity, enhance selectivity, and mitigate off-target effects. Nevertheless, biological systems frequently exploit planar aromatic structures as functional elements for molecular recognition and stability and, consistently, many natural products retain planar aromatic scaffolds that have evolved to interact efficiently with biological membranes and protein surfaces.^69,70^ Indeed, planar aromatic compounds such as resveratrol and curcumin can adopt membrane-associated orientations that favor insertion into, or association with, the hydrophobic region of the bilayer.^69,70^ Out-of-plane substituents would be expected to create steric clashes with neighboring acyl chains, increasing the free energy cost of insertion and reducing both the thermodynamic favorability and the kinetic rate of membrane partitioning. This interpretation is consistent with the well-established observation that cholesterol, whose planar tetracyclic ring system allows tight packing with phospholipid tails, is one of the most efficient membrane-intercalating small molecules in biology. The protoberberine alkaloid series studied here effectively samples a gradient of molecular planarity within an otherwise conserved scaffold, and the data indicate that increasing deviation from planarity is associated with progressively weaker membrane interactions. Thus, while planarity may present challenges for drug-like physicochemical properties, it remains a powerful structural feature in nature for mediating specific molecular interactions and biological activity.

In conclusion, these findings provide a rational framework for understanding how Brb and its analogues interact with and permeate the lipid bilayer via passive diffusion and provide a foundation for structural optimization in this protoberberine structural class. These insights may guide the development of next-generation analogues with enhanced membrane affinity and potentially improved cellular uptake, addressing one component of the bioavailability limitations of berberine that currently restricts the clinical translation of this compound.

## Experimental Section

### Preparation of Large Unilamellar Vesicles (LUVs)

LUVs were prepared using a lipid mixture consisting of 65% (mol) 1,2-dioleoyl-*sn*-glycero-3-phosphocholine (DOPC, Avanti Polar Lipids), 33% sphingomyelin (SM, Sigma-Aldrich), 1% cholesterol (CHOL, Sigma-Aldrich), and 1% ganglioside GM1 (Avanti Polar Lipids), as previously described^7,71,72^ and known to induce lipid phase segregation.^7,72,73^ The lipids were dissolved in a chloroform/methanol solution (2:1), and the organic solvent was then removed by vacuum evaporation (Univapo 150H, UniEquip) for at least 3 h. The resulting lipid films were hydrated with bidistilled water at 60 °C for 1 h, reaching a final lipid concentration of 2 mg/mL and forming multilamellar vesicles (MLVs). These MLVs were then extruded 17 times through polycarbonate membranes with 100 nm pores using a mini-extruder (Avanti Polar Lipids) maintained at 60 °C to form LUVs. After cooling to room temperature, LUVs were stored at 4 °C and used within 1 week.

### Fluorescence Quenching and Anisotropy of TMA-DPH-Labeled LUVs Incubated with Protoberberine Alkaloids

All eight PBAs used in this work, including Brb, were purchased from DBA Italy as powders (distributed on behalf of MedChemExpress). All investigated compounds are >98% pure, as certified by the manufacturer (MedChemExpress) via HPLC or LC–MS analysis provided in the Supporting Information (Figures S4–S11). Their formula strings are reported in Table S1. Stock solutions were prepared by dissolving the powders in dimethyl sulfoxide (DMSO, Merck) at a concentration of 10 mM and stored at −20 °C until use. 1-(4-Trimethylammoniumphenyl)-6-phenyl-1,3,5-hexatriene *p*-toluenesulfonate (TMA-DPH, Thermo Fisher Scientific) was dissolved at 1 mM in methanol and added to the lipid mixture to obtain a probe:lipid molar ratio of 1:300. LUVs were then prepared at 2 mg/mL as described above, diluted with bidistilled water to 0.3 mg/mL, and incubated with increasing concentrations of PBAs as quenchers (0 to 500 μM) at 25 °C for 15 min in the dark. The fluorescence emission spectra and fluorescence anisotropy values (*r*) of the resulting samples were acquired at 25 °C from 380 to 550 nm (excitation 355 nm) and at 430 nm (excitation 355 nm), respectively, using a 3 × 3 mm black-walled quartz cell on an Agilent Cary Eclipse spectrofluorometer (Agilent Technologies) equipped with a thermostated cell holder attached to an Agilent PCB 1500 water Peltier system. *F*_0_/*F* values were plotted versus PBA concentration and fitted with the extended Stern–Volmer equation to account for contributions from both dynamic and static quenching mechanisms:

1 $$\frac{F_{0}}{F}=\left(1+K_{\mathrm{SV}}\times \left[\mathrm{PBA}\right]\right)\exp\left(K_{\mathrm{ST}}\times \left[\mathrm{PBA}\right]\right)$$

where *F*_0_ and *F* are the fluorescence intensities at 430 nm in the absence and presence of PBAs, respectively, [PBA] is the concentration of the quencher, and *K*_SV_ is the dynamic Stern–Volmer constant describing collisional quenching and *K*_ST_ is the static quenching constant, with the static quenching contribution modeled through the sphere-of-action term as an exponential dependence on quencher concentration.^46^

*r* values were plotted versus PBA concentration and fitted with

2 $$r=r_{b}+\frac{\Delta r_{\max}\times \left[PBA\right]}{k_{app}+\left[PBA\right]}+s\times \left[PBA\right]$$

where *r*_b_ represents the baseline anisotropy of TMA-DPH incorporated in the LUVs membrane in the absence of PBAs, Δ*r*_max_ is the maximal binding-induced anisotropy change, *k*_app_ is the apparent dissociation constant, and *s* is the slope describing the linear dependence of anisotropy on PBA concentration, accounting for linear or nonspecific contributions.

### Interaction Kinetics of DPH-Labeled LUVs Incubated with Protoberberine Alkaloids

1,6-Diphenyl-1,3,5-hexatriene (DPH, Merck) was dissolved at 1 mM in ethanol and added to the lipid mixture to obtain a probe:lipid molar ratio of 1:300. LUVs were then prepared at 2 mg/mL as described above, diluted with bidistilled water to 0.3 mg/mL, and incubated with 30 μM PBAs as quenchers, at 25 °C for 0–4000 s in the dark. The fluorescence emission values at 430 nm (excitation 355 nm) were acquired at 25 °C, using a 3 × 3 mm black-walled quartz cell on the Agilent Cary Eclipse spectrofluorometer described above. The acquired fluorescence emission values were normalized to a value of 1000 in the absence of PBAs, plotted versus time, and fitted with a double exponential curve:

3 $$F\left(t\right)=Q_{b}+\Delta Q_{\mathrm{fast}}\exp\left(-k_{\mathrm{fast}}\times t\right)+\Delta Q_{\mathrm{slow}}\exp\left(-k_{\mathrm{slow}}\times t\right)$$

where *F*(*t*) is the fluorescence emission at time *t*, *Q*_b_ is the baseline quenching at long times, Δ*Q*_fast_ and *k*_fast_ describe the amplitude and rate constant, respectively, of the rapid quenching phase occurring within seconds, and Δ*Q*_slow_ and *k*_slow_ describe the corresponding parameters of the slower quenching phase occurring over minutes. The slow rate constant *k*_slow_ was used to compare the kinetics of different PBAs interacting with LUV membranes.

### Expression and Purification of HypF-N and Preparation of Toxic Type A (OAs) Oligomers

Expression and purification of the model protein HypF-N were carried out as previously described.^74^ In brief, HypF-N was expressed in *E. coli* XL10-Gold ultracompetent cells harboring the pQE30-Th/HypF-N plasmid. The cells were induced with IPTG (Thermo Fisher Scientific), purified by affinity chromatography using HIS-Select Nickel Affinity Gel (Thermo Fisher Scientific), buffer-exchanged in 5 mM acetate buffer and 2 mM dithiothreitol (DTT), pH 5.5, and concentrated at 700 μM before storage at −80 °C. Toxic type A oligomers (OAs) of HypF-N were obtained by incubating the protein at 0.5 mg/mL (48 μM, monomer equivalents) for 4 h at 25 °C in 50 mM acetate buffer, 12% (v/v) trifluoroethanol (TFE), and 2 mM DTT, pH 5.5, as previously described.^74^

### Preparation of Amyloid β-Derived Diffusible Ligands (ADDLs)

The lyophilized 42-residue amyloid β peptide (Aβ_42_, Bachem) was dissolved in 100% hexafluoro-2-isopropanol (HFIP) to a concentration of 1 mM and stored at −20 °C until use. Aβ_42_ oligomers in the form of amyloid-derived diffusible ligands (ADDLs) were obtained by evaporating HFIP under a nitrogen stream, dissolving the resulting peptide in anhydrous DMSO to 5 mM, and subsequently diluting it in ice-cold F-12 medium to a final concentration of 100 μM. This solution was finally incubated at 4 °C for 24 h, as described previously.^54^

### Fluorescence Quenching of TMA-DPH-Labeled LUVs with HypF-N OAs

TMA-DPH-labeled LUVs were prepared at 2 mg/mL as described above, diluted with bidistilled water to 0.3 mg/mL, and incubated with increasing concentrations of HypF-N OAs as a quencher (0 to 35 μM, monomer equivalents), at 25 °C for 15 min in the dark. When in the presence of PBAs, LUVs were preincubated for 15 min with 30 μM of each compound and then for 15 min with OAs at 25 °C in the dark. The fluorescence spectra of the resulting samples were acquired at 25 °C from 380 to 550 nm (excitation 355 nm) using a 3 × 3 mm black-walled quartz cell on the Agilent spectrofluorometer described above. The plots of quenching of TMA-DPH induced by OAs in LUVs in the absence of PBAs and in LUVs preincubated with 30 μM Clm, DmB, Jtr, Plm, and Rtn were analyzed with the Stern–Volmer equation:

4 $$\frac{F_{0}}{F}=1+K_{\mathrm{SV}}\times \left[\mathrm{OAs}\right]$$

where *F*_0_ and *F* are the fluorescence intensities at 430 nm in the absence and presence of OAs, respectively, and *K*_SV_ is the Stern–Volmer constant.

The corresponding plots with 30 μM Brb, Cpt, and EpB were analyzed from 7.5 μM OAs with an equation derived from the Stern–Volmer equation:

5 $$\frac{F_{0}}{F}=q+K_{\mathrm{SV}}\times \left[\mathrm{OAs}\right]$$

where *q* is the *y* intercept, and all the other parameters have the same meaning as in eq 4.

*F*_0_/*F* values at 35 μM OAs obtained from fitting the data with either eqs 4 or 5 in the absence and presence of PBAs were then plotted in a bar plot to compare the OA displacement ability from the membrane of LUVs of the PBAs. Δ­(*F*_0_/*F*) was calculated as the ratio between the *F*_0_/*F* values at 35 μM OAs in LUVs without and with each PBA. Higher Δ­(*F*_0_/*F*) values therefore indicate a stronger ability of the compound to reduce OA-induced quenching and displace OAs from the membrane.

### Far-UV CD Spectroscopy of HypF-N OAs Incubated with PBAs

OAs were formed in the absence and presence of PBAs at different molar ratios of protein:compound (1:0.25, 1:0.5, 1:1), which were added to native HypF-N before inducing oligomerization. Following their formation, the mixtures were diluted to 10 μM OAs (monomer equivalents) and 2.5–10.0 μM PBAs in 20 mM phosphate buffer, pH 7.0, 25 °C. Far-UV CD spectra were collected over the 195–250 nm wavelength range by averaging three spectra, with a data pitch of 0.1 nm, a scanning speed of 50 nm/min, and a response time of 1 s at 25 °C, using a 1 mm path-length cell on a Jasco J-810 spectropolarimeter equipped with a thermostated cell holder connected to a Thermo Haake C25P water bath.^75^ All spectra were truncated at HT > 700 V, blank-subtracted, and normalized to mean residue ellipticity per residue ([θ]_res_) using

6 $${[\theta ]}_{\mathrm{res}}=\frac{\theta }{10\times N_{\mathrm{res}}\times \mathrm{Optical}\;\mathrm{path}\times \left[\mathrm{Protein}\right]}$$

where *θ* is the ellipticity in mdeg units, *N*_res_ is the number of amino acid residues, Optical path is the path-length of the cell in cm units, and [*Protein*] is the protein concentration in M units.

### Anilinonaphthalene-1-sulfonate (ANS) Fluorescence Assay

10 μM HypF-N OAs (monomer equivalents) were preincubated with 0–50 μM PBAs in 100 mM potassium phosphate buffer, pH 7.0, for 15 min at 25 °C and then ANS was added at a final concentration of 100 μM. Fluorescence emission spectra were acquired at 25 °C between 435 and 650 nm (excitation at 380 nm), immediately after addition of the dye, using a 3 × 3 mm black-walled quartz cell and the Agilent Cary Eclipse spectrofluorometer described above. ANS spectra were also recorded in the presence of 0–50 μM PBAs in the absence of OAs, in order to verify whether these compounds could interact directly with ANS and thus exclude any interference in the subsequent measurements on OAs. A spectrum recorded with 10 μM OAs without ANS was subtracted from each corresponding spectrum obtained with ANS and OAs, to subtract the oligomer scattering contribution.

### Cell Culture

Authenticated human neuroblastoma SH-SY5Y cells (CRL-2266, ATCC) were grown in a culture medium containing Dulbecco’s Modified Eagle’s Medium (DMEM; D8537, Sigma-Aldrich) and Nutrient Mixture F-12 Ham (N4888, Sigma-Aldrich). The medium was supplemented with 10% fetal bovine serum (FBS; F2442, Sigma-Aldrich), 1.0 mM glutamine (G7513, Sigma-Aldrich), and 1.0% penicillin and streptomycin solution (P0781, Sigma-Aldrich). Cultures were maintained in a 5.0% CO_2_ humidified atmosphere at 37 °C and grown until 80% confluence for a maximum of 20 passages. Cells were routinely tested to ensure that they were free from mycoplasma contamination.

### Measurement of Intracellular Calcium Levels

Undifferentiated SH-SY5Y cells were seeded onto glass coverslips placed in 24-well plates at a density of 60,000 cells per well. After 24 h, cells were pretreated for 10 min with PBAs at increasing concentrations (from 0.0009 to 30 μM). Following pretreatment, ADDLs were added to the culture medium at a concentration of 0.5 μM (monomer equivalents) for another 10 min, together with a 5 μM X-Rhod-1 AM red fluorescent probe (X14210, Thermo Fisher Scientific). The calcium-derived fluorescence was then detected immediately by a TCS SP8 scanning confocal microscopy system (Leica Microsystems), equipped with a diode-pumped solid-state (DPSS) laser. For each sample, a series of 10 optical sections (1024 × 1024 pixels), 1.0 μm in thickness, was acquired throughout the cell depth using a Leica Plan Apo 63× oil immersion objective and then projected as a single composite image by superimposition. Acquisition parameters including detector gain, pinhole diameter, and laser power were kept constant across each experiment. Images were analyzed using the ImageJ software. A normalized response (*R*) was then calculated according to the following equation:

7 $$R=\frac{F_{S}-F_{ADDLs}}{F_{U}-F_{ADDLs}}\times 100$$

where *F*_S_ is the fluorescence measured in samples treated with ADDLs and PBAs, *F*_ADDLs_ is the fluorescence in cells exposed to ADDLs without PBAs, and *F*_U_ is the fluorescence in untreated cells (set as 100%).

Plots of normalized response (*R*) values versus PBA concentration ([PBA]) were then fitted with the Hill equation:

8 $$R=\frac{E_{\max}}{1+{\left(\frac{{\mathrm{EC}}_{50}}{\left[\mathrm{PBA}\right]}\right)}^{n}}\times 100$$

where *E*_max_ represents the maximal effect of each PBA, EC_50_ is the PBA concentration producing 50% of the maximal response, and *n* is the Hill coefficient, which describes the steepness of the dose–response curve.

In another experiment, cells were treated for 20 min with 10 μM PBA in the absence of ADDL treatment. Intracellular calcium levels were then measured as described above.

### MTT Reduction Assay

Undifferentiated SH-SY5Y cells were seeded onto 96-well plates at a density of 15,000 cells per well. After 24 h, cells were treated with PBAs at 10 μM. After another 24 h, the culture medium was removed, cells were washed with PBS, and the MTT solution was added to the cells for 4 h. The formazan product was solubilized with cell lysis buffer (20% sodium dodecyl sulfate (SDS), 50% *N*,*N*-dimethylformamide, pH 4.7) for 1 h. The absorbance values of blue formazan at 595 nm were determined through a Multiskan FC Microplate Photometer (Thermo Scientific). Cell viability was expressed as the percentage of MTT reduction as compared to untreated cells (taken as 100%).

## Supplementary Material





## Data Availability

The raw data that support the findings of this study are available from the corresponding author [Fabrizio.chiti@unifi.it] upon reasonable request.

## Notes

Table S1 reports PBA formula strings and CAS No. Complete dataset, including log­(*EC*_50_) and *E*_max_ values is provided in a separate file (CSV)
Scatter plots correlating physicochemical descriptors versus membrane interaction, kinetics, anisotropy, fluorescence quenching, and intracellular calcium assay log­(*EC*_50_) values (Figure S1); scatter plots showing linear correlations between membrane affinity, kinetics, ordering, and oligomer displacement (Figure S2); confocal scanning microscopy images and free calcium level histograms alongside MTT reduction assays for intrinsic cytotoxicity evaluation (Figure S3); RP-HPLC chromatogram of berberine chloride (Figure S4); LC–MS analytical report of columbamine chloride (Figure S5); coptisine chloride (Figure S6), demethyleneberberine chloride (Figure S7), and epiberberine chloride (Figure S8); RP-HPLC chromatogram of jatrorrhizine chloride (Figure S9), and palmatine chloride (Figure S10); LC–MS analytical report of rotundine (Figure S11); and protoberberine alkaloids molecular formula strings (SMILES) for all investigated compounds (PDF)

## Abbreviations

ADDLs: Aβ_42_ amyloid-derived diffusible ligands
ANS: anilinonaphthalene-1-sulfonate
Brb: berberine
CHOL: cholesterol
Clm: columbamine
Cpt: coptisine
DmB: demethyleneberberine
DMEM: Dulbecco’s Modified Eagle’s Medium
DOPC: 1,2-dioleoyl-*sn*-glycero-3-phosphocholine
DPH: 1,6-diphenyl-1,3,5-hexatriene
DPSS: diode-pumped solid state
*EC*_50_: concentration resulting in half protection
*E*_max_: value of maximum protection
EpB: epiberberine
Δ­(*F*_0_/*F*): reduction in OA-induced quenching
FBS: fetal bovine serum
GM1: ganglioside GM1
HFIP: hexafluoro-2-isopropanol
HT: high tension
Jtr: jatrorrhizine
*k*_fast_: fast rate constant
*k*_slow_: slow rate consntat
*K*_ST_: static quenching constant
*K*_SV_: Stern–Volmer constant
log­(*K*_ow_): logarithm of the octanol/water partition coefficient
LUVs: large unilamellar vesicles
MLVs: multilamellar vesicles
MTT: 3-(4,5-dimethylthiazol-2-yl)-2,5-diphenyltetrazolium bromide
*N*_HB_: number of hydrogen-bond donors and acceptors
OAs: HypF-N oligomers of type A
PBAs: protoberberine alkaloids
Plm: palmatine
*r*: fluorescence anisotropy
*R*: normalized response
Rtn: rotundine
SM: sphingomyelin
[*θ*]_res_: mean residue ellipticity per residue
TFE: trifluoroethanol
TMA-DPH: 1-(4-trimethylammoniumphenyl)-6-phenyl-1,3,5-hexatriene p-toluenesulfonate
