# Supplementary material for: Structure–Activity Relationship Study of Protoberberine Alkaloids Reveals Molecular Planarity as a Key Determinant of Membrane Interaction and Protection from Misfolded Protein Oligomers
Source: J Med Chem. 2026 Jul 27;69(15):19338–54. doi: 10.1021/acs.jmedchem.6c01682 (PMC13492292; doi:10.1021/acs.jmedchem.6c01682)
Supplement: Supplementary file 1 [file jm6c01682_si_002.pdf]

Supporting Information

Structure-activity relationship study of protoberberine alkaloids reveals molecular planarity as a key determinant of membrane interaction and protection from misfolded protein oligomers

Silvia Errico[1], Alessandra Bigi[1], Giulia Fani[1], Monica Ambrosino[1], Erwan Galmiche[1,2], Francesco Bemporad[1], Michele Vendruscolo[3], Benedetta Mannini[1,3], Fabrizio Chiti[1*]

[1]Department of Experimental and Clinical Biomedical Sciences, Section of Biochemistry, University of Florence, 50134 Florence, Italy

[2]Chimie ParisTech - PSL University, 75005 Paris, France

[3]Centre for Misfolding Diseases, Yusuf Hamied Department of Chemistry, University of Cambridge, CB2 1EW Cambridge, UK

[*]Corresponding author: Fabrizio Chiti (fabrizio.chiti@unifi.it)

Contents

Figure S1. Scatter plots correlating physicochemical descriptors versus membrane interaction, kinetics, anisotropy, fluorescence quenching, and intracellular calcium assay *log(EC$_{50}$)* values *(Page S2)*.

Figure S2. Scatter plots showing linear correlations between membrane affinity, kinetics, ordering, and oligomer displacement *(Page S3)*.

Figure S3. Confocal scanning microscopy images and free calcium level histograms alongside MTT reduction assays for intrinsic cytotoxicity evaluation *(Page S4)*.

Figure S4. RP-HPLC chromatogram of Berberine chloride *(Page S5)*.

Figure S5. LC-MS analytical report of Columbamine chloride *(Page S6)*.

Figure S6. LC-MS analytical report of Coptisine chloride *(Page S7)*.

Figure S7. LC-MS analytical report of Demethyleneberberine chloride *(Page S8)*.

Figure S8. LC-MS analytical report of Epiberberine chloride *(Page S9)*.

Figure S9. RP-HPLC chromatogram of Jatrorrhizine chloride *(Page S10)*.

Figure S10. RP-HPLC chromatogram of Palmatine chloride *(Page S11)*.

Figure S11. LC-MS analytical report of Rotundine *(Page S12)*.

Table S1. Protoberberine alkaloids molecular formula strings *(Page S13)*.

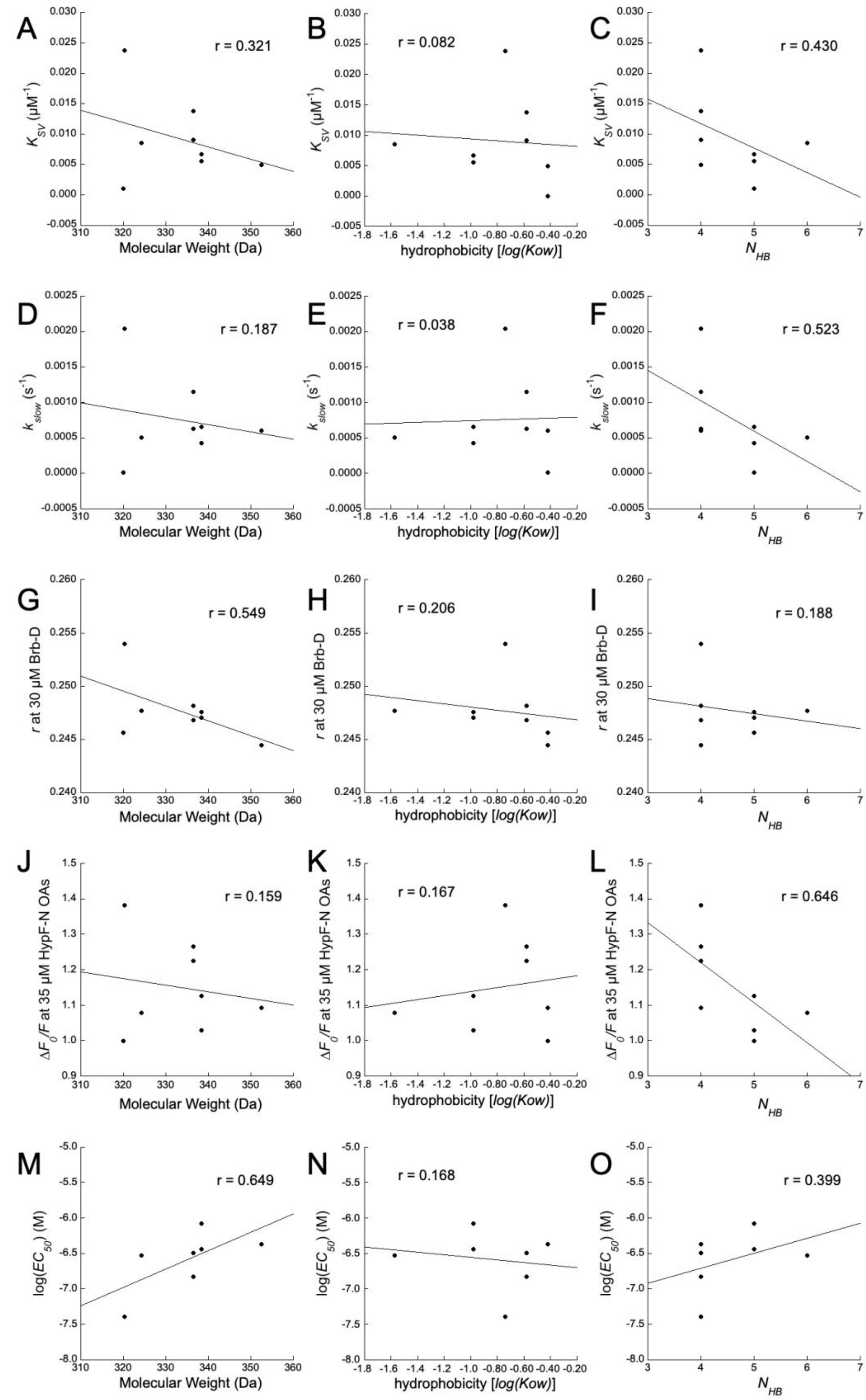

Figure S1. Classical physicochemical descriptors do not readily explain membrane interaction of protoberberine alkaloids. Scatter plots correlating K_{SV} values obtained from TMA-DPH-labelled LUVs (**A-C**), k_{slow} values derived from interaction kinetics with DPH-labelled LUVs (**D-F**), fluorescence anisotropy (*r*) values of TMA-DPH-labelled LUVs with 30 µM PBAs (**G-I**), variation of fluorescence quenching ($\Delta F_0/F$) at 35 µM HypF-N OAs with PBAs (**J-L**), and $log(EC_{50})$ values obtained from intracellular Ca^{2+} assays (**M-O**) *versus* molecular weight (left panels), hydrophobicity expressed as $log\ (K_{ow})$ (central panels) and number of hydrogen bonds (right panels) of the eight investigated PBAs. Straight lines through the data points representing the linear regression fits and linear correlation coefficients (*r*) are reported.

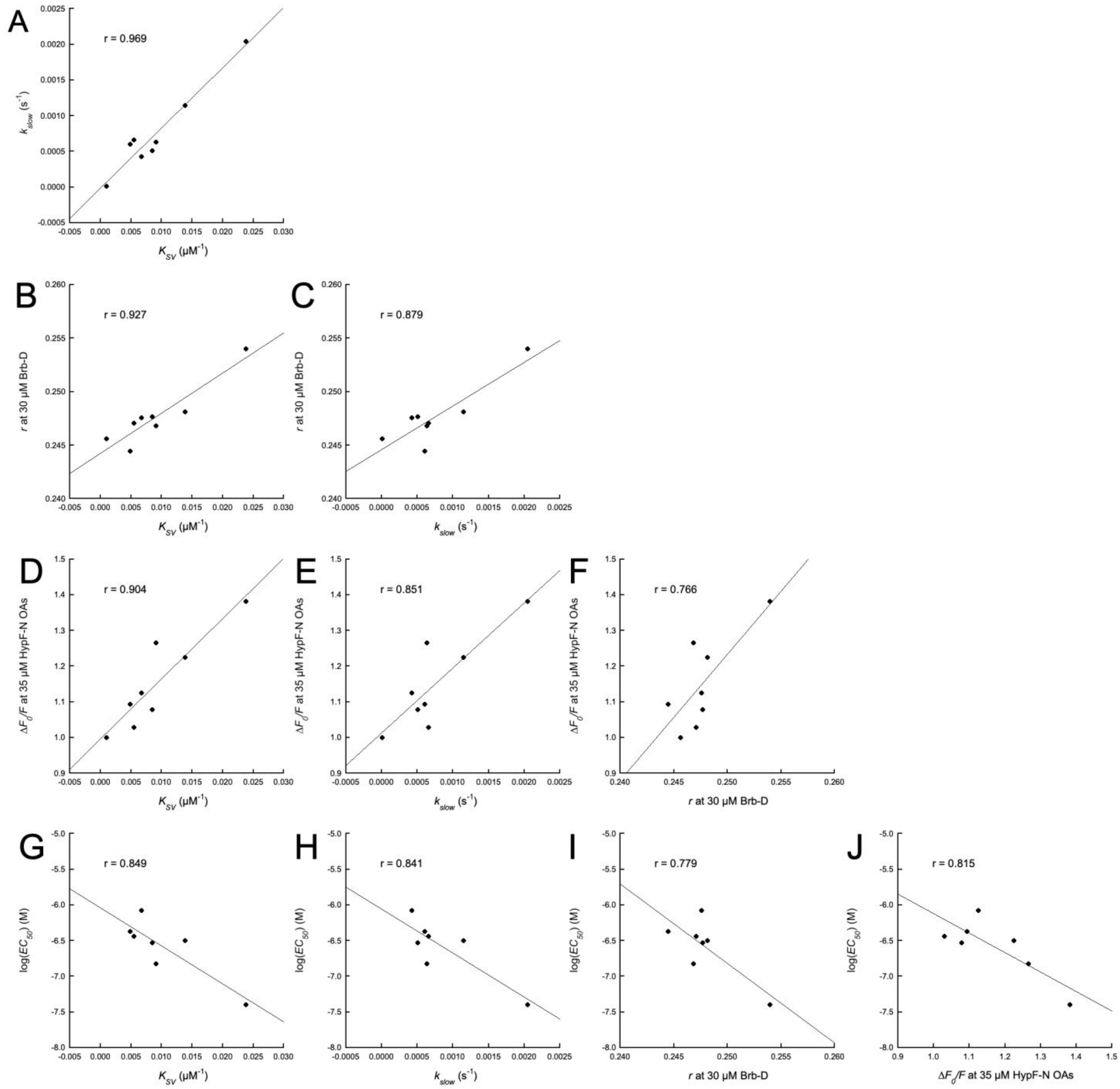

Figure S2. Membrane affinity, kinetics, ordering and oligomer displacement are strongly coupled. Scatter plots showing the linear correlations between the rate constant of the slow phase (k_{slow}) and the Stern-Volmer constant K_{SV} **(A),** the fluorescence anisotropy (*r*) at 30 µM PBAs and K_{SV} **(B)** or k_{slow} **(C)**, the ratio of fluorescence intensity variation ($\Delta F_0/F$) at 35 µM HypF-N OAs and K_{SV} **(D)**, k_{slow} **(E)**, or *r* at 30 µM PBAs **(F)**, the $log(EC_{50})$ derived from cellular toxicity assays and K_{SV} **(G)**, k_{slow} **(H),** *r* at 30 µM PBAs **(I)** or $\Delta F_0/F$ at 35 µM HypF-N OAs **(J).** Straight lines through the data points representing the linear regression fits and linear correlation coefficients (*r*) are reported.

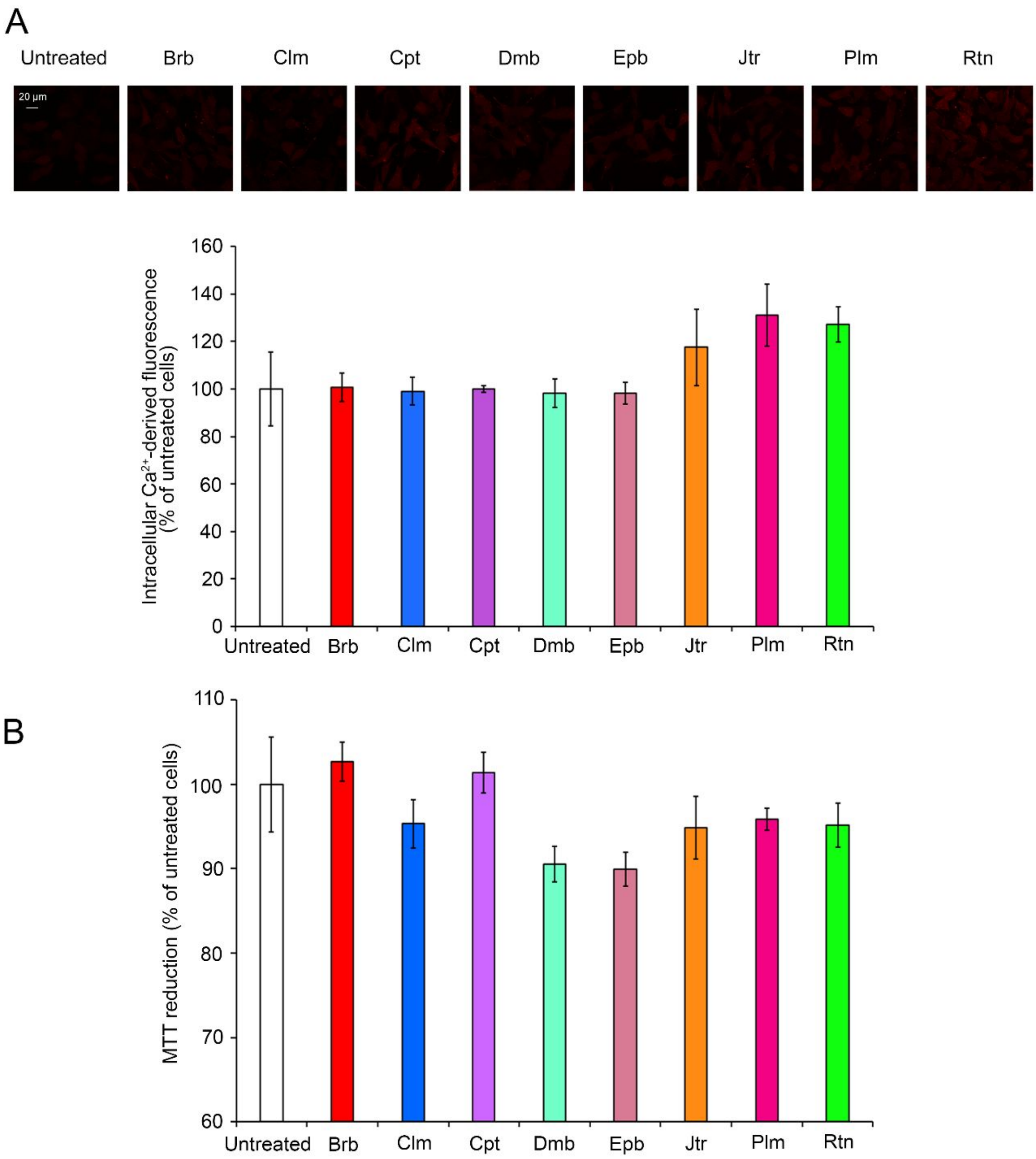

Figure S3. Protoberberine alkaloids do not induce intrinsic cytotoxicity or calcium dysregulation. (A) Representative confocal scanning microscopy images of intracellular free Ca^{2+} levels in SH-SY5Y cells treated with the indicated PBA (10 µM, 20 min). Cells were then loaded with the X-Rhod-1 AM red fluorescent probe. The histogram reports the semi-quantitative analysis of Ca^{2+}-derived fluorescence. **(B)** MTT reduction in SH-SY5Y cells treated with the indicated PBA (10 µM, 24 h). Data are expressed as mean ± SEM (n = 3).

Sample Name:	CYX44570-016-11-P	Acquired By:	WXT3564
CAS NO.:	633-65-8	Sample Set Name:	20250408_HPLC140
Vial:	1:E,4	Acq. Method Set:	HY_1154_11_H01RS01_AB_C1_35min
Injection #:	1	Processing Method:	HY_1154_9_35min_210nm
Injection Volume:	5.00 ul	Channel Name:	2998 Ch1 210nm@4.8nm
Run Time:	35.0 Minutes	Remark:	HY-1154_11-20250408 1mg/ml
Date Acquired:	4/9/2025 5:13:52 AM CST	Column Number:	A-RP-1030 50%ACN
Date Processed:	4/9/2025 10:08:14 AM CST	Processed By:	WXT3564/group_leader

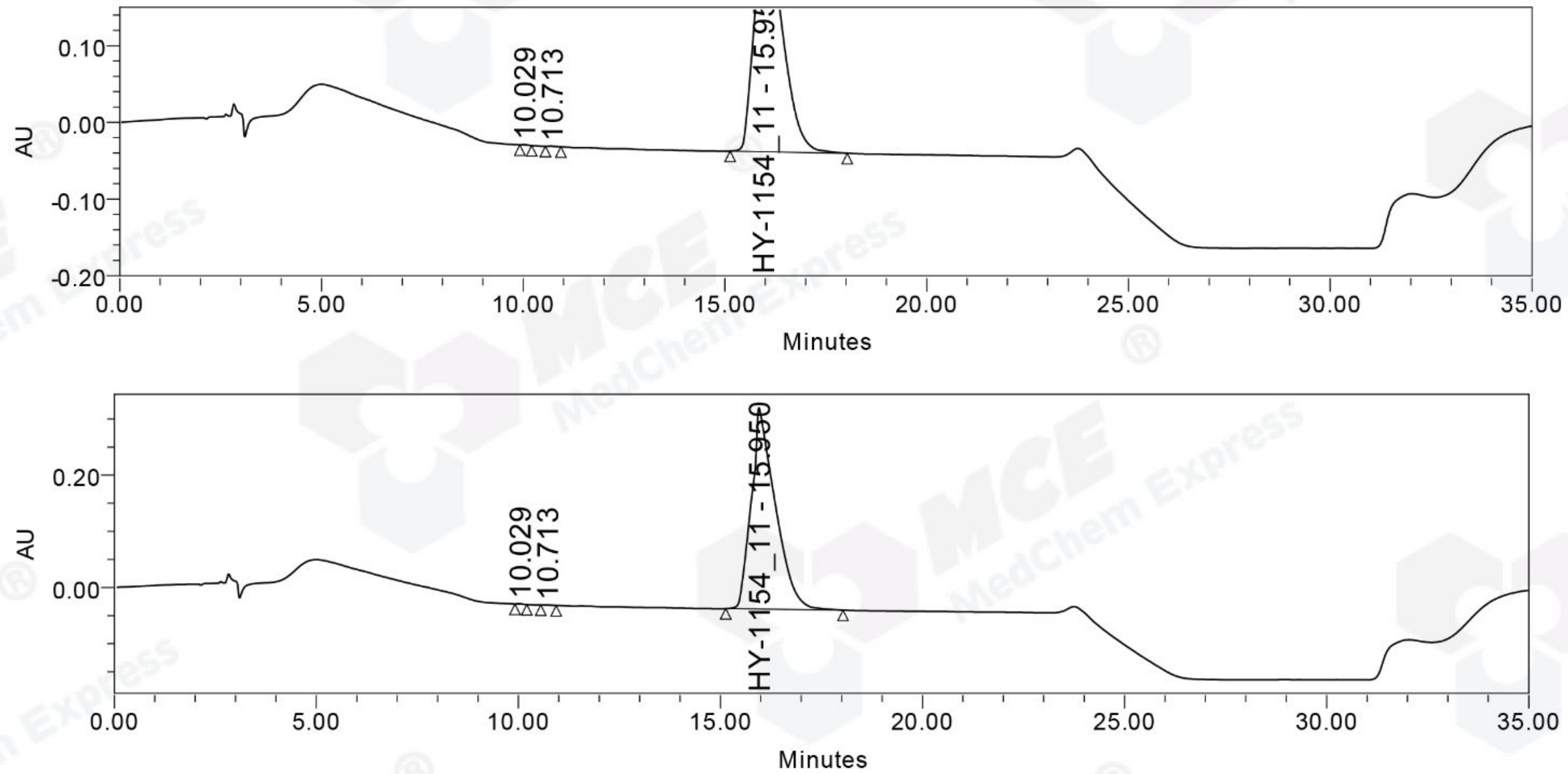

Figure S4. RP-HPLC chromatogram of Berberine chloride (CAS 633-65-8) indicating chemical identity and purity determined by UV integration.

```
File E:\ChemStation\1\DATA\2025\SW-05\2025-05-27\BIZ2025-526-95-UPLC-MS-10-064694.D
Injection Date     : 28-May-25, 03:05:11              Tgt Mass(EZX) :
Sample Name        : BIZ2025-526-95                        Location : P1-E-06
Acq. Operator      : LiZhuping                                  Inj : 1
Spec. Reported     : MS Integration                    Inj Volume : 0.8 ul
Acq. Method        : E:\ChemStation\1\Methods\1-POS-UPLC3.0MIN(1.2)(150-1000).M
Analysis Method    : E:\CHEMSTATION\1\METHODS\1-POS-UPLC3.0MIN(1.2)(150-1000).M
CAS NO.      : 1916-10-5
Method Info : Mobile Phase: A: water(0.01%TFA) B:ACN(0.01%TFA)
              Gradient: 5% to 95%B within 2.0 min
              Flow Rate :1.2ml/min
              Column :Shim-pack Scepter C18-120, 3.0*33mm,3um
              Oven Temperature : 45C
```

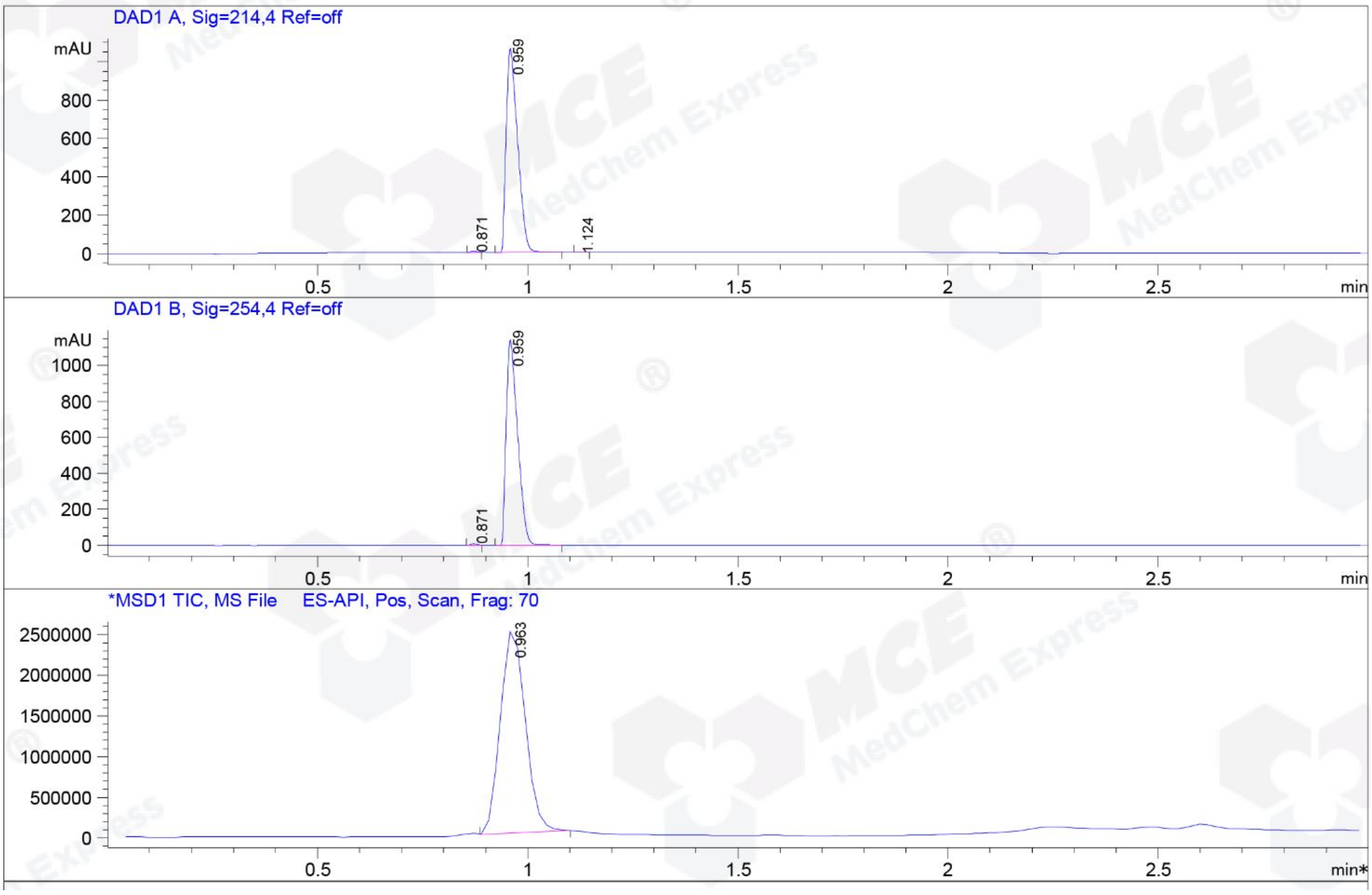

Figure S5. LC-MS analytical report of Columbamine chloride (CAS 1916-10-5) indicating chemical identity and a high purity profile determined by UV integration.

File E:\ChemStation\1\DATA\2024\2024-12\2024-12-10\CPK2024-D10-114136-UPLC-MS-10-050805.D
Injection Date : 10-Dec-24, 11:24:23 Tgt Mass(EZX) :
Sample Name : CPK2024-D10-114136 Location : P1-D-02
Acq. Operator : LY_2278 Inj : 1
Spec. Reported : MS Integration Inj Volume : 0.8 ul
Acq. Method : E:\ChemStation\1\Methods\1-POS-UPLC3.0MIN(1.2)(150-1000).M
Analysis Method : E:\CHEMSTATION\1\METHODS\1-POS-UPLC3.0MIN(1.2)(150-1000).M
CAS NO. : 6020-18-4 WalkUp method: '1-POS-3MIN' Project: CPK2024
Method Info : Mobile Phase: A: water(0.01%TFA) B:ACN(0.01%TFA)
Gradient: 5% to 95%B within 2.0 min
Flow Rate :1.2ml/min
Column :Shim-pack Scepter C18-120, 3.0*33mm,3um
Oven Temperature : 45C

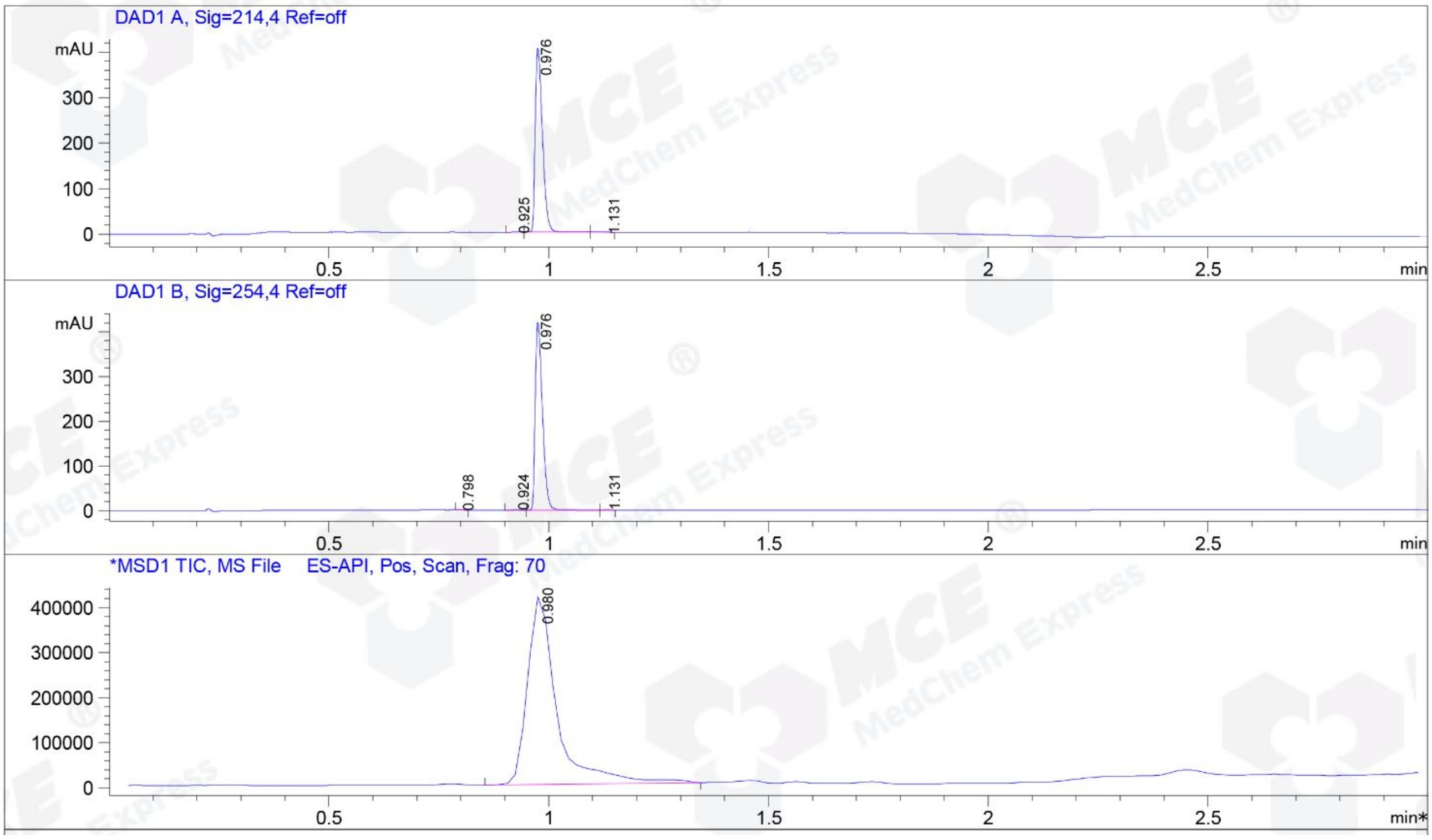

Figure S6. LC-MS analytical report of Coptisine chloride (CAS 6020-18-4) indicating chemical identity and a high purity profile determined by UV integration.

File E:\ChemStation\1\DATA\2025\SW-05\2025-05-27\BIZ2025-526-103-UPLC-MS-10-064645.D
Injection Date : 27-May-25, 20:20:13 Tgt Mass(EZX) :
Sample Name : BIZ2025-526-103 Location : P1-A-01
Acq. Operator : LiZhuping Inj : 1
Spec. Reported : MS Integration Inj Volume : 0.8 ul
Acq. Method : E:\ChemStation\1\Methods\1-POS-UPLC3.0MIN(1.2)(150-1000).M
Analysis Method : E:\CHEMSTATION\1\METHODS\1-POS-UPLC3.0MIN(1.2)(150-1000).M
CAS NO. : 16705-03-6 WalkUp method: '1-POS-3MIN' Project: BIZ2025
Method Info : Mobile Phase: A: water(0.01%TFA) B:ACN(0.01%TFA)
Gradient: 5% to 95%B within 2.0 min
Flow Rate :1.2ml/min
Column :Shim-pack Scepter C18-120, 3.0*33mm,3um
Oven Temperature : 45C

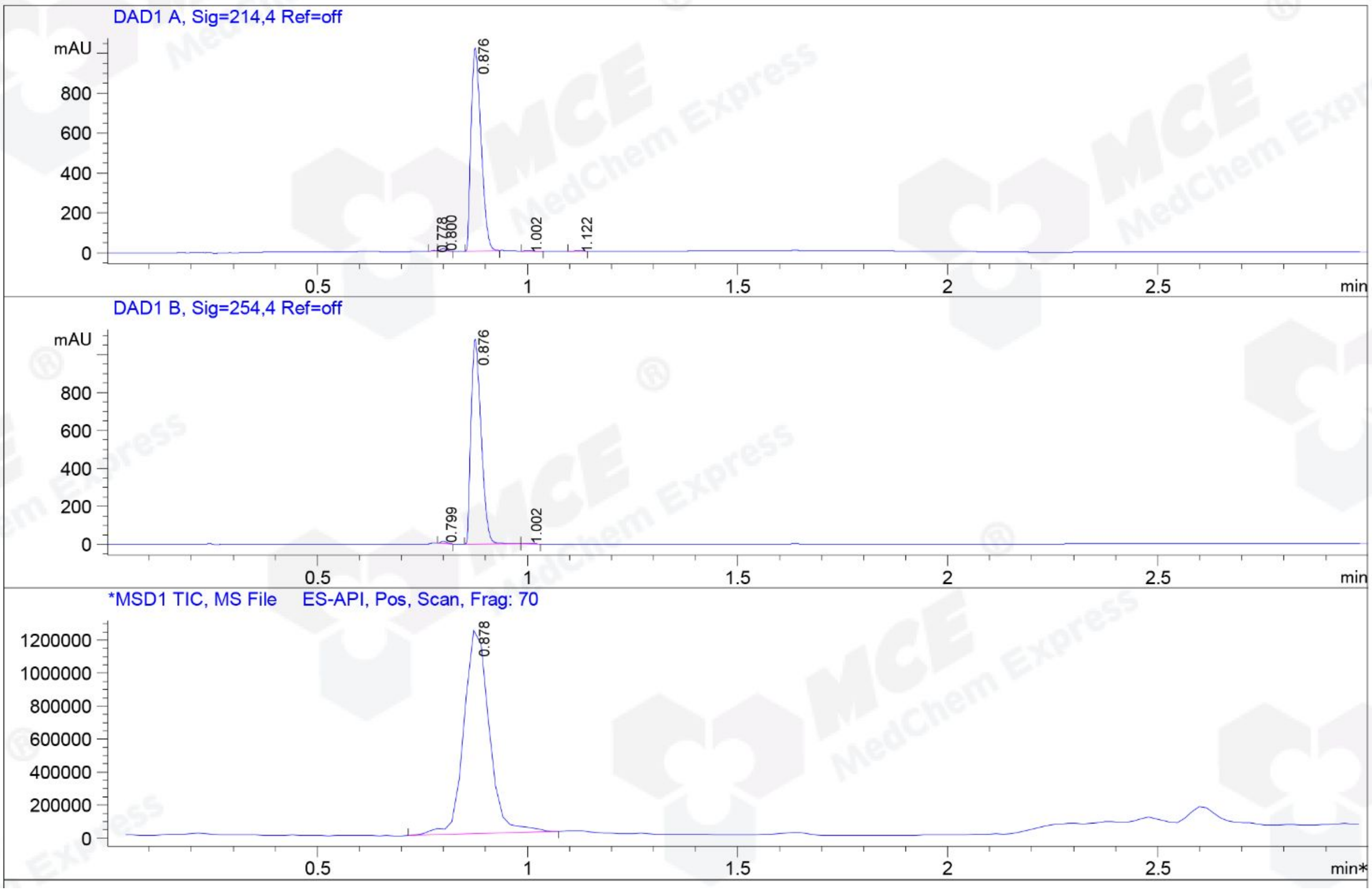

Figure S7. LC-MS analytical report of Demethyleneberberine chloride (CAS 16705-03-06) indicating chemical identity and a high purity profile determined by UV integration.

```
File E:\ChemStation\1\DATA\2025\SW-05\2025-05-15\CPK2025-0514-398008-UPLC-MS-10-062965.D
Injection Date    : 15-May-25, 14:18:08            Tgt Mass(EZX) :
Sample Name       : CPK2025-0514-398008                      Location : P1-C-05
Acq. Operator     : LiZhuping                                      Inj : 1
Spec. Reported    : MS Integration                      Inj Volume : 0.8 ul
Acq. Method       : E:\ChemStation\1\Methods\1-POS-UPLC3.0MIN(1.2)(150-1000).M
Analysis Method   : E:\CHEMSTATION\1\METHODS\1-POS-UPLC3.0MIN(1.2)(150-1000).M
CAS NO.      : 889665-86-5
Method Info : Mobile Phase: A: water(0.01%TFA) B:ACN(0.01%TFA)
              Gradient: 5% to 95%B within 2.0 min
              Flow Rate :1.2ml/min
              Column :Shim-pack Scepter C18-120, 3.0*33mm,3um
              Oven Temperature : 45C
```

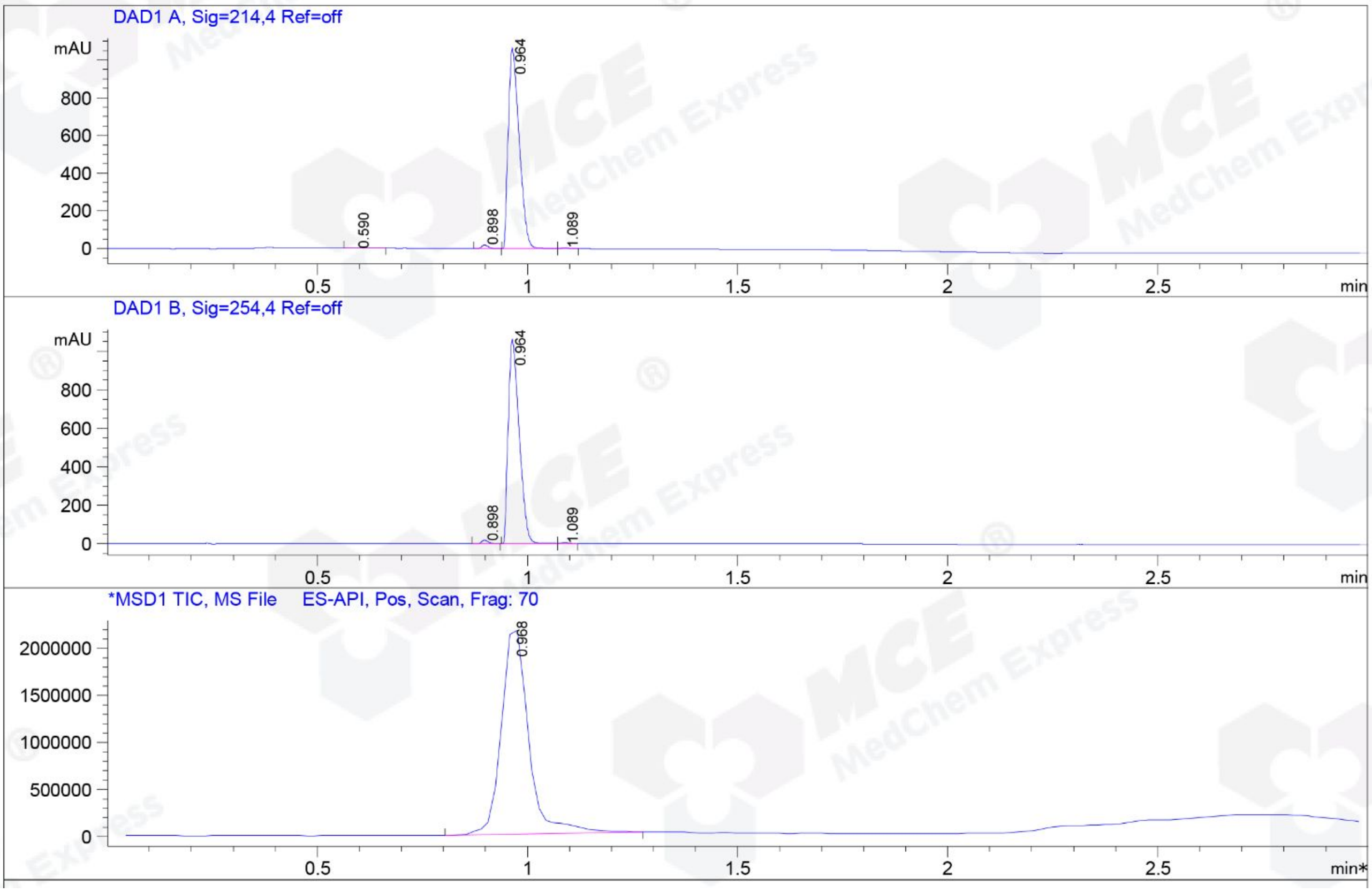

Figure S8. LC-MS analytical report of Epiberberine chloride (CAS 889665-86-5) indicating chemical identity and a high purity profile determined by UV integration.

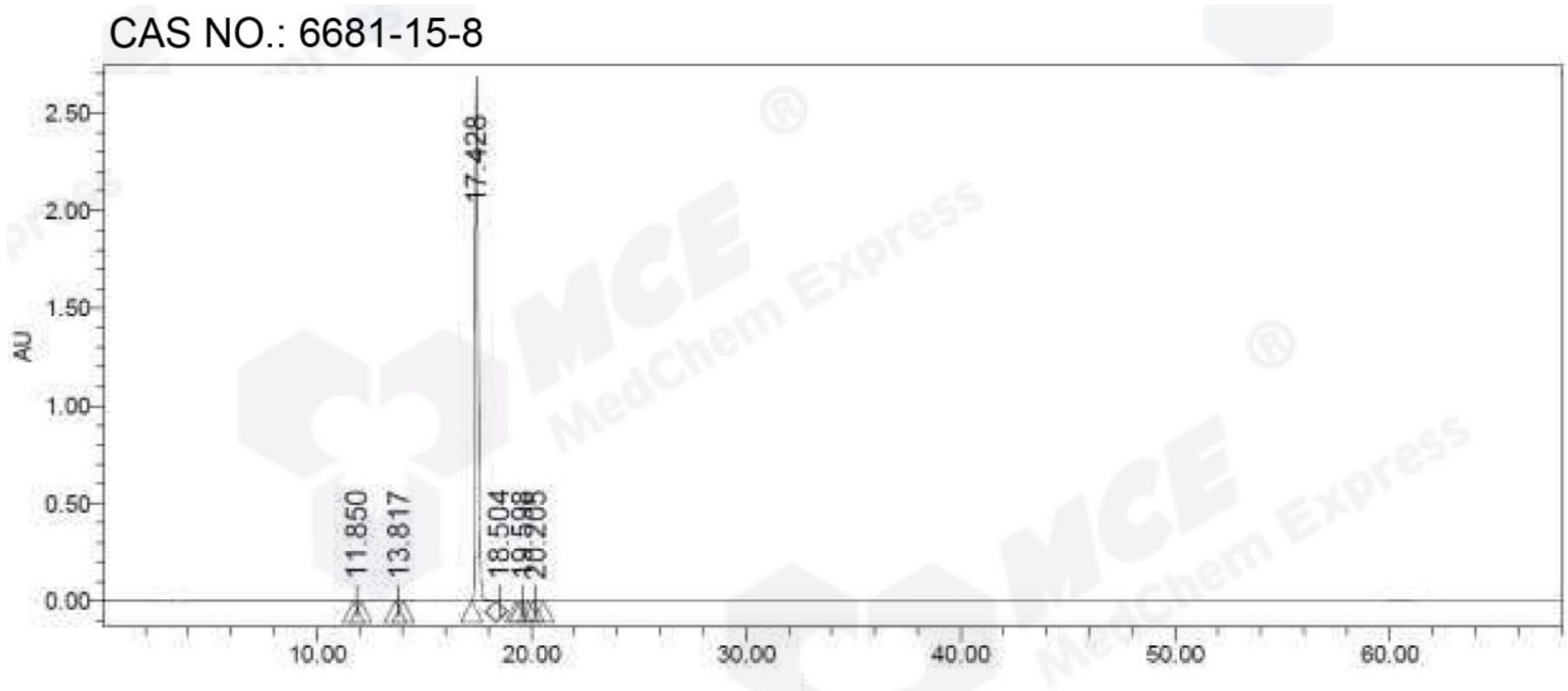

Figure S9. RP-HPLC chromatogram of Jatrorrhizine chloride (CAS 6681-15-8) indicating chemical identity and purity determined by UV integration.

Chromatogram and Results

Injection Details

Injection Name:	**CPK2025-1021-263644**	*Run Time (min):*	**30.00**
Vial Number:	**BC3**	*Injection Volume:*	**2.00**
Injection Type:	**Unknown**	*Channel:*	**UV_VIS_1**
CAS NO.:	**10605-02-4**	*Wavelength:*	**345**
Instrument Method:	**HY-N0110**	*Bandwidth:*	**4**
Processing Method:	**3D Quantitative**	*Dilution Factor:*	**1.0000**
Injection Date/Time:	**17/Nov/25 19:01**	seq.update_operator	**JLW1433**
seq.creation_operator	**JLW1433**	instMeth.creation_operator	**JLW1433**
injection.dilution_factor	**1.0000**	rdf.update_operator	**HDE2815**
instMeth.creation_time	**17/Nov/2025 17:53**		
chm.rawpath	**chrom://hy637/XVault/HPLC-56/1925/20251117.seq/567.smp/UV_VIS_1.channel/UV_VIS_1.chm**		

Chromatogram

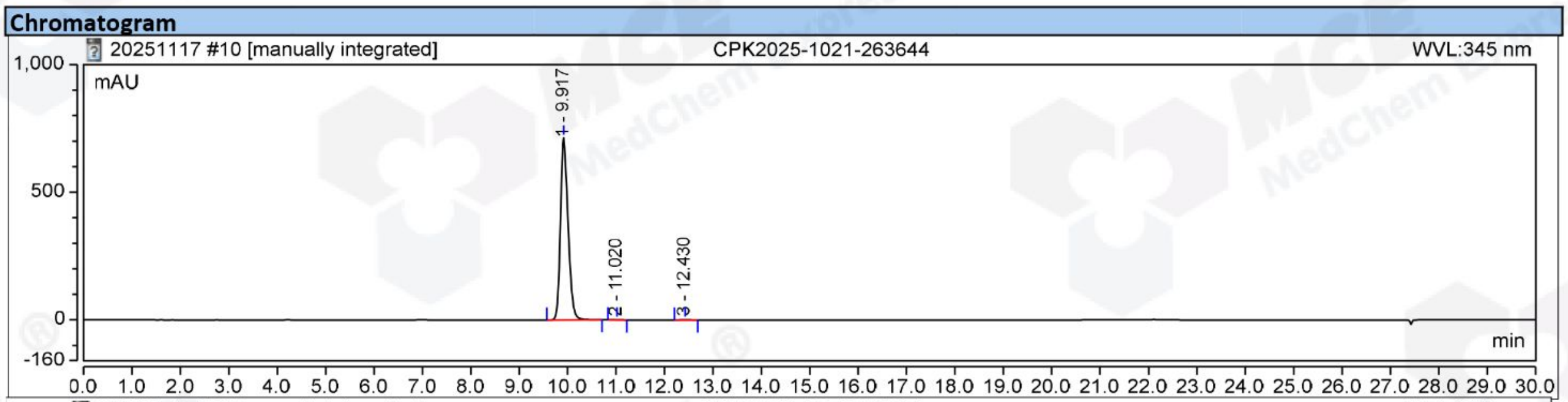

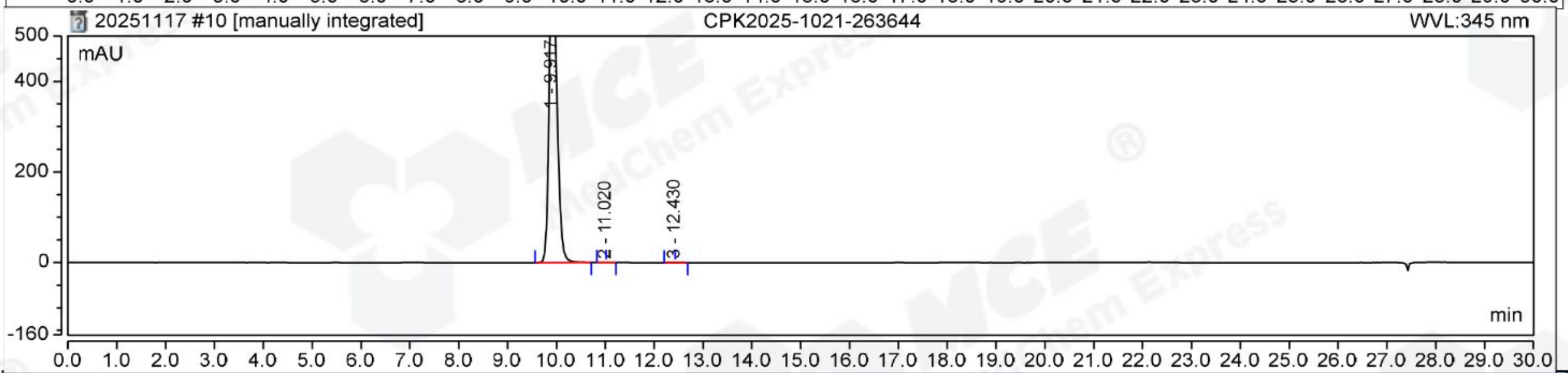

Integration Results

No.	Peak Name	Retention Time min	Area mAU*s	Height mAU	Relative Area %	Resolution (SM)	Plates (EP)	Asymmetry (EP)
1		9.917	7637.7642	713.525	99.8788	4.13	20827	1.27
2		11.020	1.3820	0.168	0.0181	5.64	35815	0.97
3		12.430	7.8883	0.710	0.1032	n.a.	28516	1.09
**Total:**			**7647.0345**	**714.403**	**100.0000**		**85158**	

Figure S10. RP-HPLC chromatogram of Palmatine chloride (CAS 10605-02-4) indicating chemical identity and purity determined by UV integration.

File E:\ChemStation\1\DATA\2024\2024-08\2024-08-27\CPK2024-826-150993-UPLC-MS-10-043040.D
Injection Date : 27-Aug-24, 12:13:23 Tgt Mass(EZX) :
Sample Name : CPK2024-826-150993 Location : P1-B-09
Acq. Operator : LY_2278 Inj : 1
Spec. Reported : MS Integration Inj Volume : 0.8 ul
Acq. Method : E:\ChemStation\1\Methods\1-POS-UPLC3.0MIN(1.2)(150-1000).M
Analysis Method : E:\CHEMSTATION\1\METHODS\1-POS-UPLC3.0MIN(1.2)(150-1000).M
CAS NO. : 483-14-7 WalkUp method: '1-POS-3MIN' Project: CPK2024
Method Info : Mobile Phase: A: water(0.01%TFA) B:ACN(0.01%TFA)
Gradient: 5% to 95%B within 2.0 min
Flow Rate :1.2ml/min
Column :Shim-pack Scepter C18-120, 3.0*33mm,3um
Oven Temperature : 45C

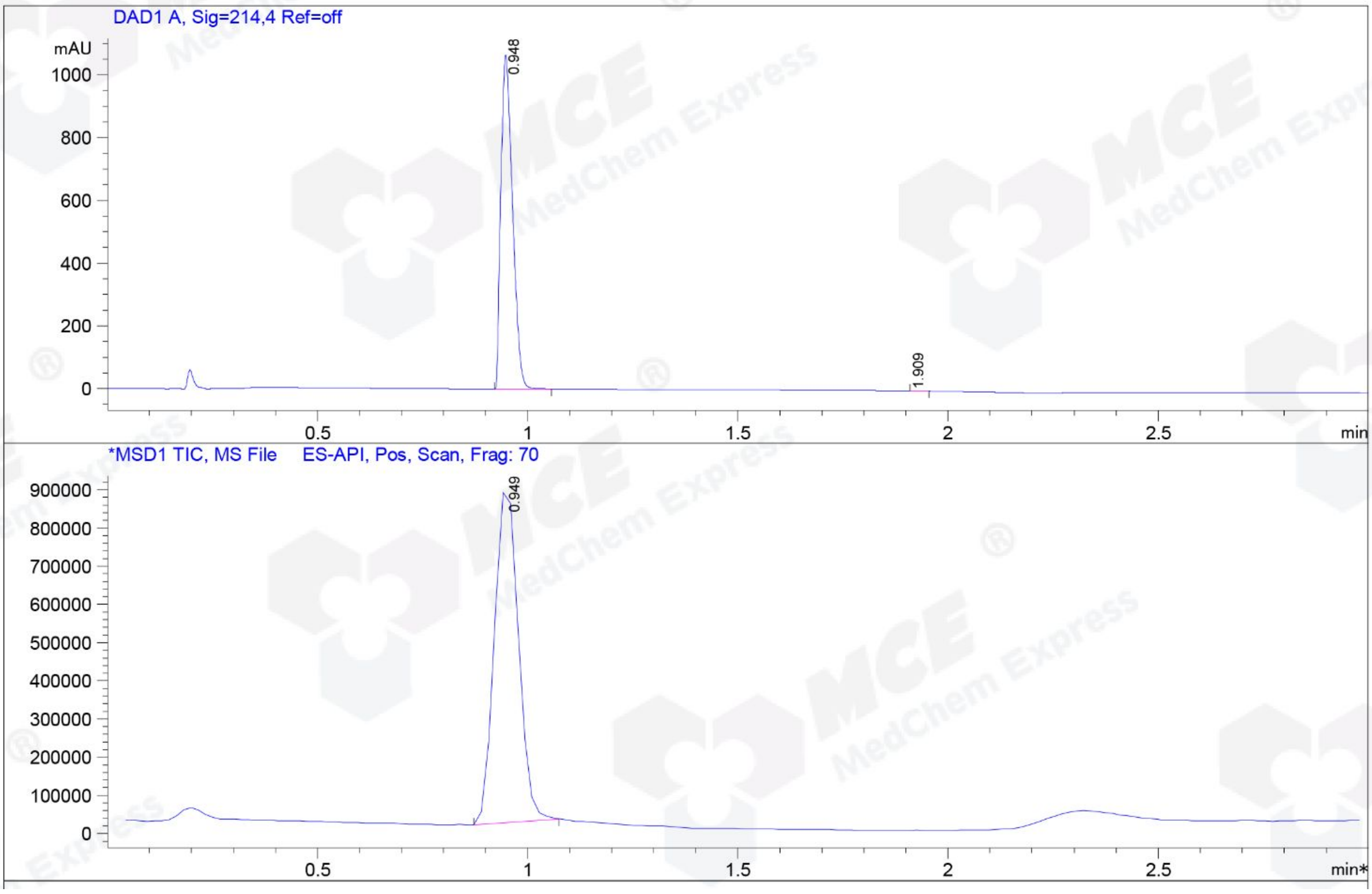

Figure S11. LC-MS analytical report of Rotundine (CAS 483-14-7) indicating chemical identity and a high purity profile determined by UV integration.

Table S1. PBAs Formula strings

Compound	SMILE Formula strings	CAS No.
**Berberine (chloride)**	COC1=C(OC)C2=C[N+]3=C(C(C(CC3)=C4)=CC5=C4OCO5)C=C2C=C1.[Cl-]	633-65-8
**Columbamine (chloride)**	COC1=C(OC)C2=C[N+]3=C(C4=CC(O)=C(OC)C=C4CC3)C=C2C=C1.[Cl-]	1916-10-5
**Coptisine (chloride)**	C1(C(CC[N+]2=C1C=C(C=C3)C(C4=C3OCO4)=C2)=C5)=CC6=C5OCO6.[Cl-]	6020-18-4
**Demethyleneberberine (chloride)**	COC1=C(OC)C2=C[N+]3=C(C4=CC(O)=C(O)C=C4CC3)C=C2C=C1.[Cl-]	16705-03-6
**Epiberberine (chloride)**	COC1=C(OC)C=C2C(CC[N+]3=C2C=C(C=C4)C(C5=C4OCO5)=C3)=C1.[Cl-]	889665-86-5
**Jatrorrhizine (chloride)**	COC1=C(OC)C2=C[N+]3=C(C4=CC(OC)=C(O)C=C4CC3)C=C2C=C1.[Cl-]	6681-15-8
**Palmatine (chloride)**	COC1=C(OC)C2=C[N+]3=C(C4=CC(OC)=C(OC)C=C4CC3)C=C2C=C1.[Cl-]	10605-02-4
**Rotundine**	COC1=CC=C2C(CN3CCC4=CC(OC)=C(OC)C=C4[C@]3([H])C2)=C1OC	483-14-7
